# From Seaweed to Corrosion Inhibitor: A Multi-Method Validation of λ‑Carrageenan as a Non-Toxic Inhibitor under Acidic Hydrodynamic Conditions

**DOI:** 10.1021/acsomega.6c05701

**Published:** 2026-08-17

**Authors:** Ana Flávia Martins Rodrigues, Larissa Caruso da Eira, Fábio Jr Ferreira da Silva Henrique, Eduardo Alencar de Souza, Rodrigo da Silva, Juliana Oliveira Pereira, Maria José O. C. Guimarães, José Antônio da Cunha Ponciano Gomes, Luana Barros Furtado

**Affiliations:** † Departamento de Processos Orgânicos (Escola de Química), Universidade Federal do Rio de Janeiro (UFRJ), Av. Athos da Silveira Ramos, 149, Cidade Universitária, Rio de Janeiro, RJ 21941-909, Brasil; ‡ Instituto de Química, 28125 Universidade Federal do Rio de Janeiro, Av. Athos da Silveira Ramos, 149, Cidade Universitária, Rio de Janeiro, RJ 21941-909, Brasil; § Laboratório de Corrosão (Programa de Engenharia Metalúrgica e de Materiais/COPPE), Universidade Federal do Rio de Janeiro (UFRJ), Av. Horácio Macedo, 2030, Cidade Universitária, Rio de Janeiro, RJ 21941-914, Brasil; ∥ Instituto de Macromoléculas Professora Eloisa Mano, Universidade Federal do Rio de Janeiro (UFRJ), Av. Horácio Macedo, 2030, Cidade Universitária, Rio de Janeiro, RJ 21941-914, Brasil

## Abstract

Corrosive processes are responsible for direct and indirect costs in industrial systems, which can be mitigated by corrosion inhibitors. The awareness of environmental risks has motivated sustainable approaches, such as naturally derived biopolymers, due to low toxicity and inhibition potential. λ-carrageenan, a sulfated biopolymer obtained from red algae, is explored in this work as an environmentally benign corrosion inhibitor. Gravimetric analyses indicate that the addition of 1500 ppm of λ-carrageenan markedly decreases the corrosion rate of mild steel, yielding inhibition efficiencies of 87.1% at 303 K and 72.0% at 333 K. Electrochemical techniques were employed under static conditions (0 rpm) and under hydrodynamic conditions at 500 and 1500 rpm to mimic flowing conditions expected in transport pipelines, achieving 89.8% at 500 rpm. Density functional theory calculations demonstrated that both the neutral and protonated forms of λ-carrageenan exhibit favorable electronic properties, with electron density distributed across hydroxyl and sulfate functionalities that promote interaction with the steel surface. Molecular dynamics simulations further confirmed the spontaneous adsorption onto the iron substrate at 1500 ppm, with interaction energies ranging from −158 to −179 kcal/mol across the investigated temperature interval. The protective layer, enabled by multiple adsorption sites involving SO_3_H and OH groups, accounts for the high inhibition performance. Overall, the results support λ-carrageenan as a viable green inhibitor capable of providing effective protection in industrial environments.

## Graphical abstract

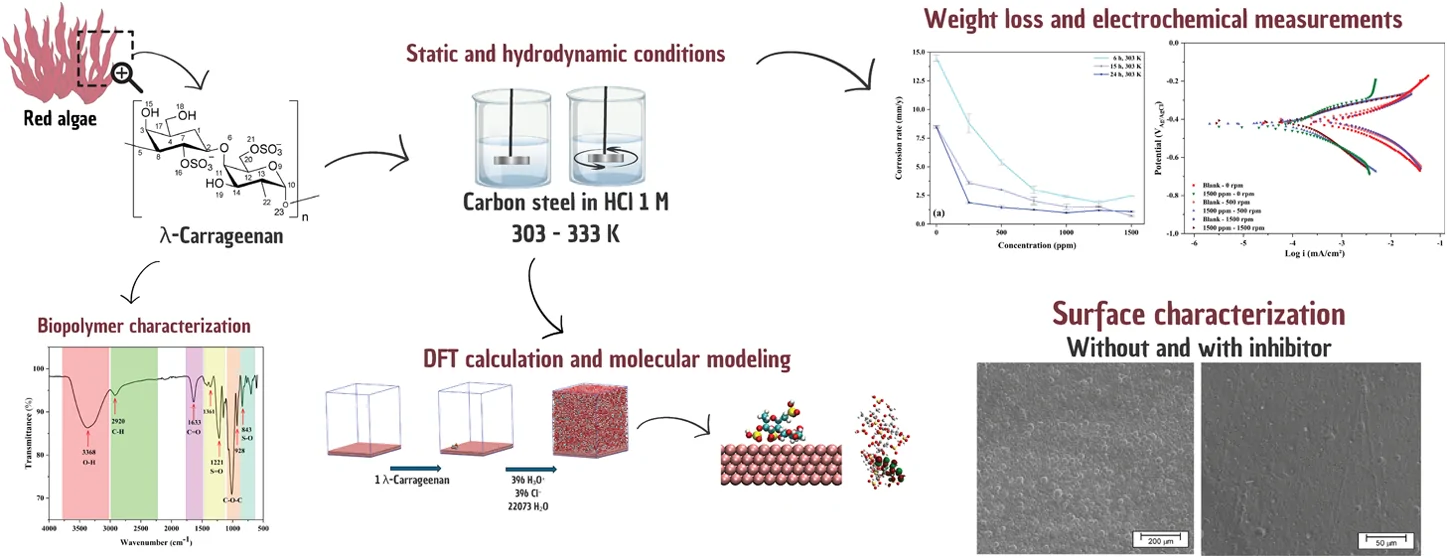

## 1 Introduction

Industrial plants frequently rely on mild steel as a construction material due to its low price, ready availability, and satisfactory properties.^1^ In parallel, acidic solutions are integral to various industrial treatments, including cleaning, pickling, and scale removal.^2^ The high exposure of mild steel to these acidic solutions leads to corrosion, causing economic losses and threatening public safety.^3^ Therefore, to control metal dissolution, industries use corrosion inhibitors. However, the use of toxic substances derived from inorganic compounds, such as heavy metals, has led to growing regulatory restrictions.^4^ In addition, some highly efficient organic compounds, such as acetylenic alcohols and thioureas, frequently reported as part of patented formulations, are responsible for health risks.^3,5,6^

In response to the need for more sustainable molecules, recent studies have focused on organic compounds with environmentally benign properties. Such compounds typically contain heteroatoms and multiple bonds, such as conjugated systems and triple bonds, which enhance their ability to adsorb onto metal surfaces.^7^ Various renewable materials have been explored as sources for inhibitor formulations, including plants,^8–10^ seeds,^11,12^ as well as extracts and polysaccharides.^1,13–15^

Biopolymer-based materials have been widely described in the literature as effective corrosion inhibitors, including starch derivatives,^16,17^ chitosan derivatives,^18,19^ pectin,^20,21^ alginate-based surfactant derivatives,^21,22^ carrageenan,^16^ and grafted guar gum.^17,18^

The inhibition of mild steel corrosion in acidic media by chitosan derivatives was investigated by Zhang et al.^19^ In that study, two new compounds were synthesized, and inhibition efficiency of up to 98.0% was achieved at 150 mg/L (24 h of immersion, 1 M HCl). In a similar approach, the inhibitory performance of pectin was reported by Fiori-Bimbi et al.^20^ A maximum efficiency of 94.2% was obtained at 2.0 g/L (318 K, 1 M HCl); however, the influence of extended immersion times was not evaluated.^20^ Alginate surfactant derivatives were examined as corrosion inhibitors by Tawfik.^21^ In that study, several compounds were synthesized, and their inhibition efficiency was shown to increase with concentration at 298 K. Among them, the copper–alginate surfactant complex (AS–Cu) presented a notable efficiency of 96.3% at 5 × 10^–3^ M in 1 M HCl.^21^ The use of ι-carrageenan for aluminum corrosion mitigation in hydrochloric acid was assessed by Fares et al.,^16^ in the presence of pefloxacin mesylate as a zwitterionic additive. Under these conditions, a maximum inhibition efficiency of 74.2% was achieved at 1600 ppm (283 K, 2 M HCl), although the exposure time was limited to 2 h.^16^ Additionally, Roy et al.^18^ demonstrated that grafted guar gum can reach inhibition efficiencies exceeding 90.0% at 500 ppm after prolonged immersion periods of up to 50 h in 1 M HCl.^18^

Given the documented corrosion-inhibition performance of biopolymers, the natural biopolymer λ-carrageenan is a promising alternative, particularly since it has not been reported previously. Carrageenan comprises a family of sulfated polysaccharides obtained from certain species of red marine algae belonging to the Rhodophyta phylum.^23^ Carrageenans are classified into six principal types: iota (ι), kappa (κ), lambda (λ), mu (μ), nu (ν), and theta (θ).^24^ The κ-, ι-, and λ-forms differ mainly in the sulfation degree of their repeating disaccharide units, containing one, two, and three sulfate ester groups, respectively, which correspond to approximate sulfate contents of 20.0%, 33.0%, and 41.0% by mass.^25^ κ-Carrageenan is primarily derived from the tropical seaweed *Kappaphycus alvarezii*, which is commercially referred to as *Eucheuma cottonii* (or simply cottonii). In turn, ι-carrageenan is mainly obtained from *Eucheuma denticulatum*, known in the trade as *Eucheuma spinosum* (or spinosum). To produce the commercial κ and ι forms, these seaweeds undergo alkaline extraction at high temperatures, a process that converts the biological precursors μ and ν into their respective commercial types. λ-Carrageenan, on the other hand, is extracted from various species belonging to the genera *Gigartina* and *Chondrus*. The sporophytic plants of these seaweeds produce λ-carrageenan, while the gametophytic plants generate a hybrid κ/ι type.^24^

In this work, the environmental benefits of a readily available, low-cost biopolymer were investigated as a corrosion inhibitor in an industrially relevant acidic medium. The influence of exposure time, concentration, and temperature on the biopolymer’s performance has been addressed. The chemical characteristics and stability of the inhibitor were examined using spectroscopic techniques and thermal analysis, while its protective behavior was evaluated through corrosion tests conducted under both static and flowing conditions. Microscopic surface characterization was employed to assess film formation and surface protection, and theoretical computational calculations provided molecular-level insight into the interaction between the biopolymer and the metallic surface. Notably, this study extends beyond conventional static evaluations by addressing hydrodynamic conditions, which are rarely reported in this corrosive environment, to clarify the influence of fluid flow on inhibitor adsorption, stability, and overall inhibition performance under realistic operating conditions.

## 2 Results and Discussion

### 2.1 Biopolymer Characterization

#### 2.1.1 FTIR Analysis

As shown in Figure 1, the FTIR spectrum of λ-carrageenan exhibits a broad band around 3368.49 cm^–1^, which is attributed to O–H stretching vibrations, together with a band near 2920 cm^–1^ associated with aliphatic C–H (sp^3^) stretching modes.^26,27^ A further absorption feature observed at approximately 1633 cm^–1^ can be assigned to the superposition of asymmetric C=O stretching and N–H bending vibrations.^28^ The spectrum also exhibits several bands typical of sulfated polysaccharides, including peaks at 1361 cm^–1^, 1221.52 cm^–1^, 928.42 cm^–1^, and 843.48 cm^–1^. These signals correspond to sulfate groups,^27^ ester sulfate functionalities,^27^ C–O–C vibrations of 3,6-anhydro-galactose units,^26,27^ and O–SO_3_ stretching associated with galactose-4-sulfate,^26,27^ respectively. These bands confirm the presence of oxygen and sulfur, possible adsorption centers on the substrate.

**Figure 1:**
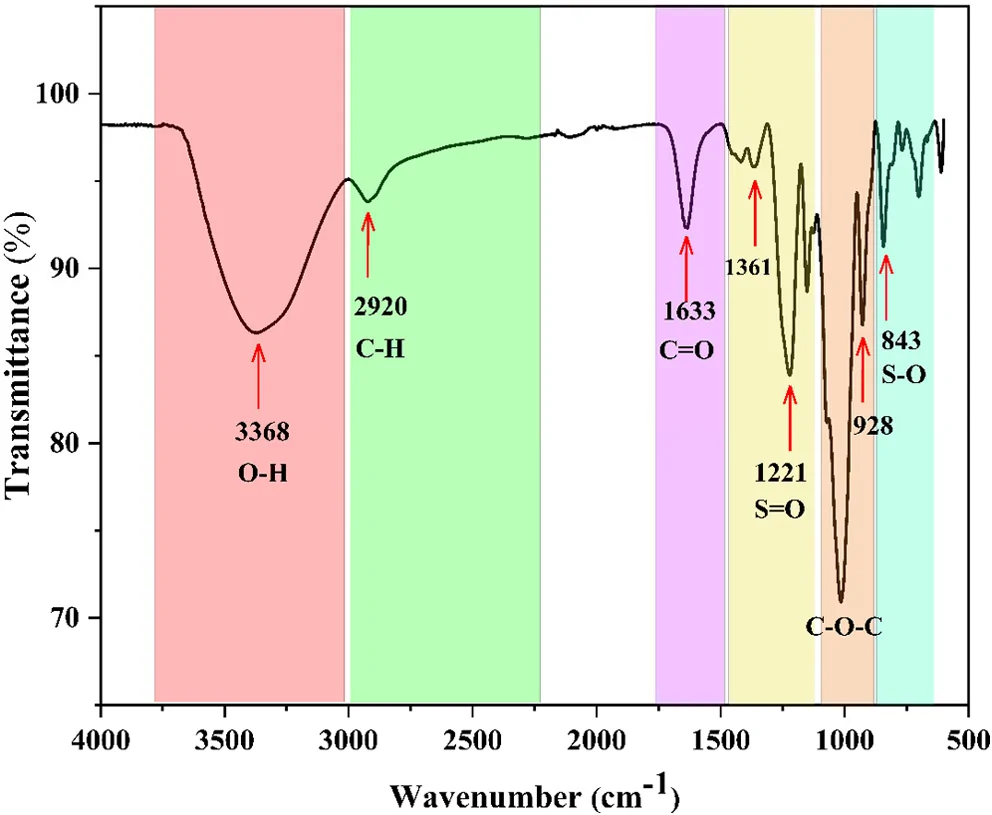
FTIR spectra of λ-carrageenan, from 600 to 4000 cm^–1^, highlighting the most essential bands associated with functional groups usually responsible for adsorption onto the metallic substrate.

Umoren et al.^29^ investigated apple-derived pectin as a corrosion inhibitor for X60 steel in 0.5 M HCl and reported comparable adsorption sites, including −OH, −COOH, and −COOCH_3_ groups. According to DFT analyses, some of these functionalities become protonated in acidic environments. As a result, the protonated biopolymer species preferentially adsorb onto cathodic regions of the X60 steel surface, thereby suppressing the hydrogen evolution reaction. In parallel, hydroxyl groups can interact with anodic sites by coordinating to vacant d orbitals on iron, thereby hindering metal dissolution.

Similarly, chitosan-based derivatives have been evaluated as corrosion inhibitors for Q235 mild steel in 1 M HCl,^19^ presenting functional groups such as −OH, −COC, −NH_2_, −C=N, and −CN.^19^ The presence of nitrogen-containing groups introduces additional adsorption centers within the polymer framework. DFT studies indicate that, under acidic conditions, certain oxygen and nitrogen atoms undergo protonation, enabling the polymer molecules to adsorb onto the metal surface via electrostatic interactions with chloride ions, characteristic of physisorption. Moreover, lone electron pairs and π electrons from nitrogen and oxygen atoms can participate in donor–acceptor interactions with iron, leading to the formation of coordination and back-donation bonds and, consequently, chemisorption.

#### 2.1.2 Thermogravimetric Analysis (TGA)

Figure 2 displays the thermogravimetric (TGA) profiles of the biopolymer. The initial mass-loss stage was observed at around 320 K and corresponded to a 14.3 ± 0.2 wt % reduction, attributed to physically adsorbed water. Comparable behavior was reported by Nandal et al.^26^ for κ-carrageenan, who observed a weight loss of 18.6 wt % from 308 to 363 K and likewise attributed this event to moisture removal. The subsequent thermal event exhibited a maximum degradation temperature (*T*_max_) of 487 K, resulting in a mass loss of 17.4 ± 0.2 wt %. Similar *T*_max_ values near 485 K were reported by Sand et al.^30^ and Mishra, Tripathy, and Behari^31^ for κ-carrageenan.^30,31^ The third decomposition stage occurred at approximately 572 K, resulting in a weight reduction of 30.8 ± 0.6 wt %, similar to the mass loss of 20.0% between 480 and 603 K reported by Nandal et al.^26^ According to Wardhana et al.,^32^ the mass decrease observed in the 443–623 K range can be attributed to the degradation of sulfonate (−SO_3_−) functional groups within the biopolymer matrix. The final decomposition resulted in a residual mass loss of 26.8 ± 4.3 wt %, occurring at 984 K. A comparable FDT close to 973 K for κ-carrageenan was previously reported by Sand et al.^30^

**Figure 2:**
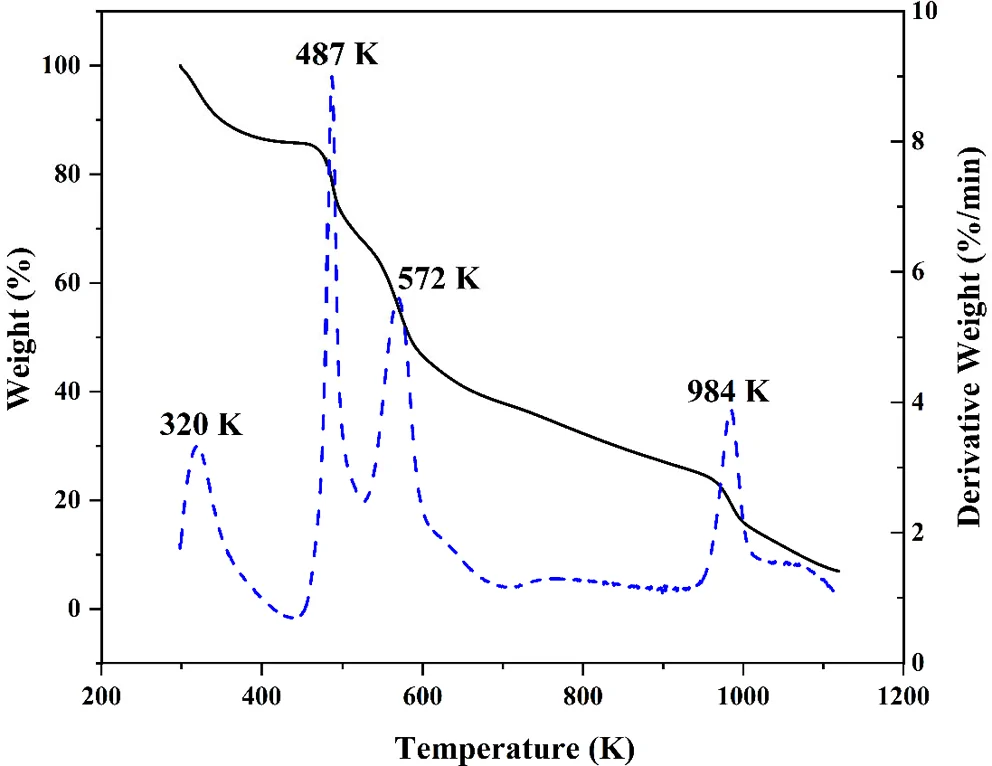
TG (black solid line) and DTG (blue dashed line) curves of λ-Carrageenan under a nitrogen atmosphere (50 mL/min) at a heating rate of 10 °C/min.

### 2.2 Weight Loss Measurements

Figure 3 and Table 1 show the corrosion rates (CR, mm/year) and efficiencies (η) of λ-carrageenan as a function of temperature (303–333 K), concentration (0–1500 ppm), and immersion time (6–24 h).

**Figure 3:**
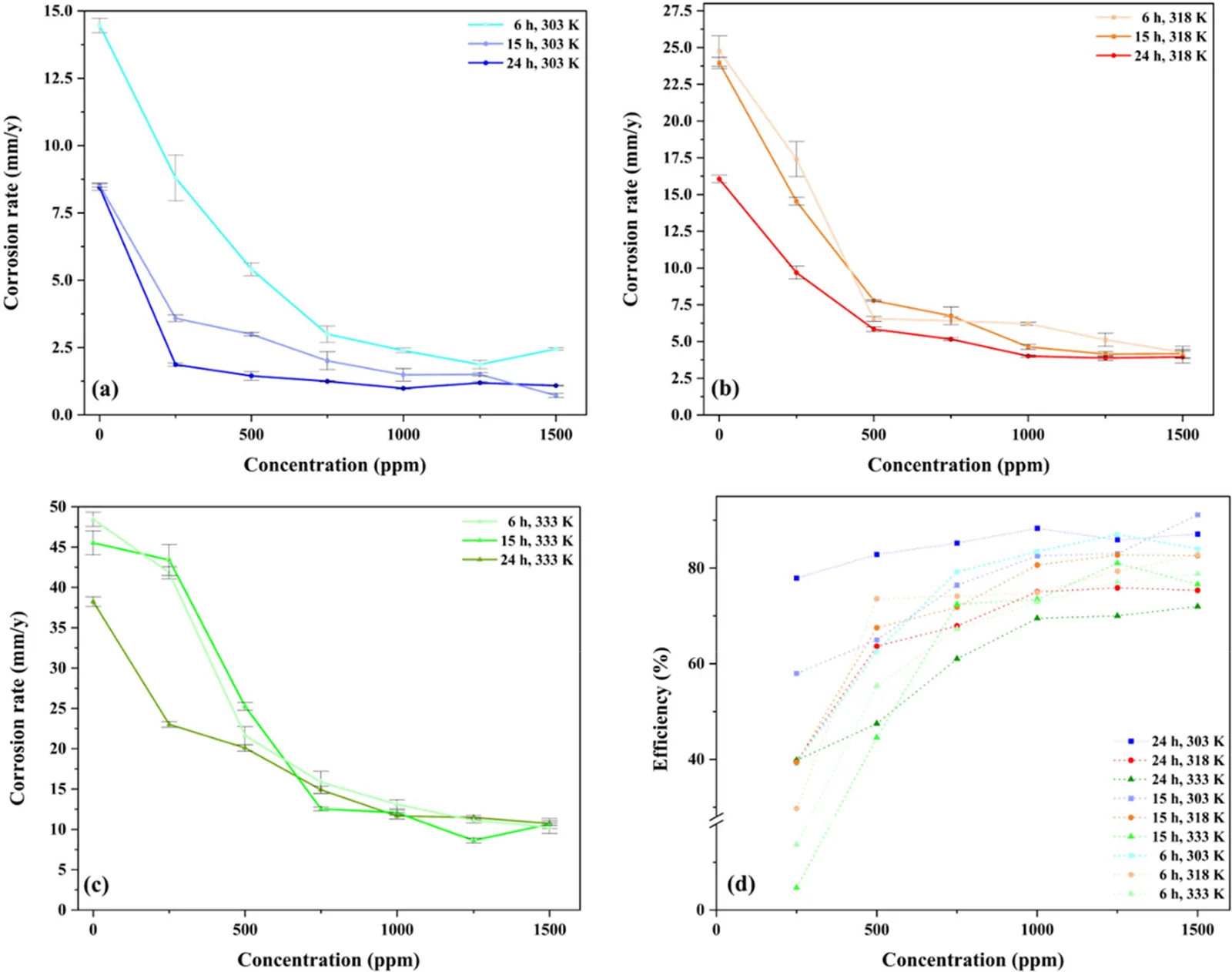
Plot of corrosion rates (a–c) and inhibition efficiency (d) as functions of λ-carrageenan concentration (250–1500 ppm), immersion time (6, 15, and 24 h), and temperature (303–333 K).

**Table 1:** Corrosion Behavior, Expressed as Corrosion Rates (CR, mm/year) and Inhibition Efficiencies (IE, %), Derived from Weight Loss Experiments Performed with and without λ-Carrageenan (250–1500 ppm), from 303 to 333 K, at 6, 15, and 24 h

		303 K		318 K		333 K	
concentration (ppm)	time (h)	IE (%)	CR (mm/year)	IE (%)	CR (mm/year)	IE (%)	CR (mm/year)
Blank	6	-	14.5 ± 0.3	-	24.8 ± 1.0	-	48.5 ± 0.9
250		39.2	8.8 ± 0.9	29.7	17.4 ± 1.2	13.7	41.8 ± 0.8
500		62.6	5.4 ± 0.2	73.6	6.6 ± 0.2	55.4	21.6 ± 1.1
750		79.2	3.0 ± 0.3	74.1	6.4 ± 0.3	67.3	15.8 ± 1.4
1000		83.5	2.4 ± 0.1	74.9	6.2 ± 0.1	72.9	13.1 ± 0.6
1250		87.1	1.9 ± 0.2	79.3	5.1 ± 0.5	77.1	11.1 ± 0.3
1500		84.1	2.5 ± 0.1	82.8	4.3 ± 0.4	78.8	10.3 ± 0.8
Blank	15	-	8.5 ± 0.1	-	24.0 ± 0.4	-	45.5 ± 1.5
250		58.0	3.6 ± 0.1	39.4	14.5 ± 0.3	4.7	43.4 ± 1.9
500		65.0	3.0 ± 0.1	67.5	7.8 ± 0.1	44.6	25.3 ± 0.5
750		76.4	2.0 ± 0.3	71.8	6.8 ± 0.6	72.4	12.6 ± 0.3
1000		82.6	1.5 ± 0.2	80.7	4.6 ± 0.2	73.5	12.1 ± 0.3
1250		83.0	1.5 ± 0.1	82.8	4.1 ± 0.2	81.0	8.6 ± 0.3
1500		91.2	0.7 ± 0.1	82.6	4.2 ± 0.3	76.6	10.7 ± 0.2
Blank	24	-	8.5 ± 0.1	-	16.1 ± 0.3	-	38.3 ± 0.6
250		78.0	1.9 ± 0.1	39.7	9.7 ± 0.5	39.8	23.0 ± 0.3
500		82.9	1.5 ± 0.2	63.7	5.8 ± 0.2	47.5	20.1 ± 0.4
750		85.2	1.3 ± 0.1	67.9	5.2 ± 0.1	61.0	14.9 ± 0.5
1000		88.4	1.0 ± 0.1	75.0	4.0 ± 0.1	69.5	11.7 ± 0.4
1250		85.9	1.2 ± 0.1	75.8	3.9 ± 0.2	70.0	11.5 ± 0.3
1500		87.1	1.1 ± 0.1	75.4	4.0 ± 0.4	72.0	10.7 ± 0.6

As shown in Figure 3(d) and Table 1, the inhibition efficiency measured after 24 h increases up to 1000 ppm at all investigated temperatures (303, 318, and 333 K). Beyond this concentration, no significant improvement is observed, suggesting the attainment of a saturation regime, which can be attributed to the biopolymer’s limited solubility in the acidic environment.^15^ A similar behavior is observed after 15 h at 318 K: the efficiency stabilizes at approximately 82.0% at 1250 ppm. For 6 h, an efficiency decrease is observed at 303 K between 1250 and 1500 ppm, probably due to the short exposure time and low temperature, which limit molecular mobility. In contrast, at 6 h, a slight efficiency increase is observed at 318 and 333 K after 1250 ppm, possibly due to greater molecular mobility at higher temperatures, which favors adsorption.

The inhibition enhancement can be explained by the increased amount of available molecules in solution to adsorb onto the metal surface as the concentration rises.^15^ The effective interaction between λ-carrageenan and the carbon steel substrate is mainly governed by functional groups containing oxygen and sulfur within its molecular structure, as evidenced by infrared analysis, which provide lone electron pairs capable of bonding with the metal surface. Comparable behavior was described by Hsissou et al.^33^ for an epoxy-based polymer containing oxygen and sulfur heteroatoms, which likewise contributed to strong adsorption and improved corrosion protection.^33^

When the influence of temperature is examined separately for each immersion period (6, 15, and 24 h), a pronounced increase in corrosion rate is observed. The kinetic energy of the system is enhanced with temperature, leading to higher molecular mobility. As a consequence, the adsorbed biopolymer layer may be destabilized, favoring partial desorption from the substrate.^16^

Under ambient conditions (303 K), the inhibition efficiency is higher at longer exposure times, as evidenced by the comparison of results obtained after 6, 15, and 24 h (Figure 3(d)). This behavior can be attributed to the macromolecular nature of carrageenan, which requires extended contact time to achieve complete surface coverage at lower temperatures. Consequently, more effective adsorption and improved inhibition performance are attained after 24 h at 303 K.^15^

However, from Table 1, when temperature and time are considered together in this analysis, a greater decline in efficiency is observed as the temperature rises at 24 h compared to 6 and 15 h. For example, at a concentration of 1000 ppm, efficiency decreases from 88.4% at 303 K to 69.5% at 333 K, a difference of approximately 20.0%. In contrast, at 6 and 15 h, the decreases are only 10.0% and 9.0%, respectively, over the same temperature range. At 1250 ppm, the efficiency difference (Δ_IE_) is even greater: Δ_IE(1250ppm)_ is only 2.0% at 15 h, while at 24 h it reaches 15.0%. This significant decline is also observed at lower concentrations, for instance, Δη_(500ppm)_ at 15 h is 20.0%, while at 24 h it is 35.0%; and Δη_(750ppm)_ is 4.0% at 15 h and 24.0% at 24 h.

At elevated temperatures, the biopolymer may exhibit increased molecular energy and mobility, which can initially favor its adsorption onto the metal surface.^34^ When coupled with shorter immersion periods, this condition may limit prolonged contact with the aggressive medium, thereby reducing molecular degradation and subsequent desorption. In contrast, at 24 h of exposure, the combined effects of extended immersion and thermal stress may induce structural rearrangement, partial decomposition, and/or desorption of λ-carrageenan from the steel surface, resulting in reduced surface protection under corrosive conditions.

Consequently, the larger Δ_IE_ observed after 24 h compared with 6 and 15 h may reflect the biopolymer’s limited stability under prolonged exposure to the acidic environment. Such behavior can be associated with partial loss of the adsorbed layer and the possible formation of smaller molecular fragments. At higher inhibitor concentrations, some surface coverage may still be maintained, which helps attenuate corrosion processes; however, this protective effect is expected to be less pronounced at lower concentrations.

The influence of concentration on each temperature as a function of immersion time is observed in Figure 3(a–c). At 303 K (Figure 3(a)), a significant decrease in CR is observed up to 750 ppm after 6 h of immersion, with further reduction up to 1250 ppm, followed by a slight loss of efficiency at the highest concentration. Higher inhibitor concentrations, therefore, result in a less pronounced additional decrease in CR. For longer immersion times, particularly 24 h, the most pronounced CR reduction is already achieved at 250 ppm, while further concentration increases lead to more gradual improvements. This observation agrees with the previous explanation based on the IE (%): at lower temperatures, lower concentrations of the macromolecule require longer immersion times to achieve effective surface coverage. To reach similar efficiencies within shorter immersion times (6 h), higher inhibitor concentrations are required.

As depicted in Figure 3(b), at 318 K, the corrosion rate (CR) decreases significantly with increasing inhibitor concentration up to approximately 500 ppm for all exposure periods, beyond which the rate of decrease diminishes, indicating the onset of a saturation regime. At 333 K (Figure 3(c)), a sharp reduction in CR is observed up to 500–750 ppm for immersion times of 6 and 15 h. In contrast, for an exposure duration of 24 h, a pronounced decrease in CR is already evident at 250 ppm, with subsequent increases in concentration producing only incremental effects. These observations imply that elevated temperatures combined with longer immersion times promote more effective surface coverage at lower inhibitor dosages, whereas shorter exposure periods require higher concentrations to achieve comparable protection. At higher inhibitor levels, deviations from ideal behavior may arise from molecular aggregation, surface rearrangement, or partial desorption, which can restrict further enhancement of surface coverage and corrosion inhibition performance.

#### 2.2.1 Temperature Effect

The influence of temperature on both the blank and the biopolymer systems was investigated using the Arrhenius relationship,^35^ as previously reported. The plot was constructed from weight loss data collected after 24 h of immersion and is shown in Figure 4. The resulting linear regressions exhibited correlation coefficients exceeding 0.98, indicating the kinetic model supports the experimental data.

**Figure 4:**
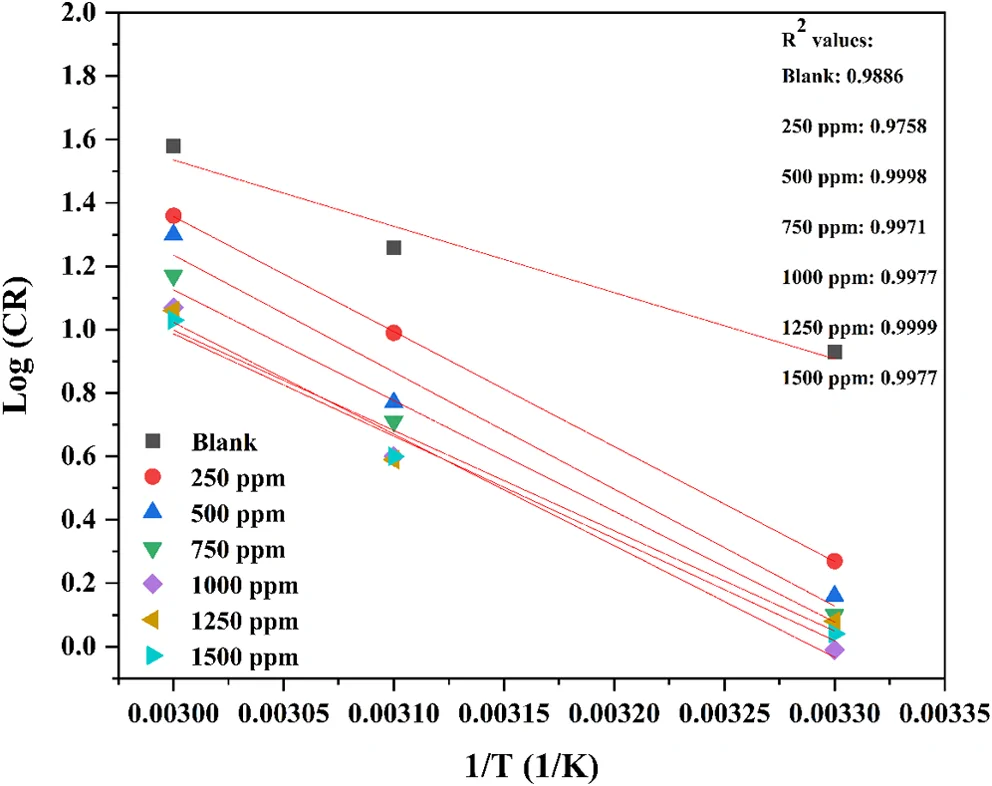
Arrhenius equation plot of logarithm of the corrosion rate (CR, mm/year) versus the reciprocal of the temperature 1/*T* (K) at 303, 318, and 333 K, for the blank and λ-carrageenan (250–1500 ppm).

Activation energy values for the inhibited system, higher than the blank, are typically indicative of physisorption, whereas lower values are generally linked to chemisorptive interactions at the metal surface.^36^ In this study, the activation energies obtained in the presence of λ-carrageenan at all evaluated concentrations exceeded the blank value, supporting the predominance of a physical adsorption mechanism.^1,37–39^ Furthermore, the standard deviation in Figure 5 shows that the activation energy values vary slightly with inhibitor concentration. A modest decrease in *E*_a_ was observed at 1250 and 1500 ppm relative to 1000 ppm, suggesting subtle changes in adsorption behavior or a reorganization of the inhibitor film at higher surface coverages.

**Figure 5:**
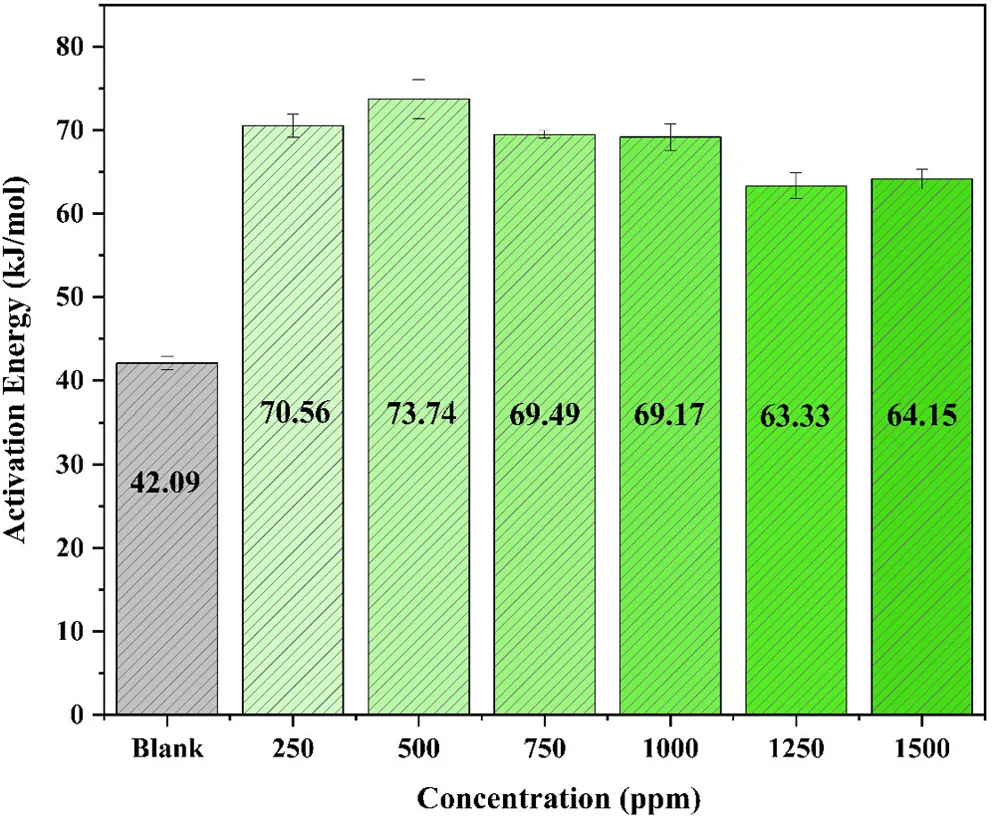
Activation energy (*E*_a_, kJ/mol) and *R* ^2^ for the blank and different concentrations of λ-carrageenan (250–1500 ppm), from Arrhenius equation plot of logarithm of the corrosion rate (CR, mm/year) versus the reciprocal of the temperature 1/*T* (K) at 303, 318, and 333 K.

The corrosion inhibitor can adsorb onto metal surfaces through various mechanisms, including physisorption, chemisorption, or the coexistence of both interactions. Literature often applies adsorption isotherms to evaluate these mechanisms and the inhibitor behavior, and four of these equations are frequently employed: Langmuir, El Awady, Flory–Huggins, and Temkin (eqs 1–4). However, in this work, adsorption isotherms and the standard Gibbs free energy of adsorption (Δ*G*°_ads_) will not be derived from corrosion data, as explained below. Instead, valuable information on adsorption will be provided through theoretical studies.

1 $$\mathrm{Langmuir}:\frac{C}{\theta }=\frac{1}{K_{\mathrm{ads}}}+C$$

2 $$\mathrm{El}\;\mathrm{Awady}:\log\left(\frac{\theta }{1-\theta }\right)=\log K+y\times \log C$$

3 $$\mathrm{Flory}‐\mathrm{Huggins}:\log\left(\frac{\theta }{C}\right)=\log K+x\times \log\left(1-\theta \right)$$

4 $$\mathrm{Temkin}:\theta =\frac{-2.303}{2a}\times \log K+\frac{-2.303}{2a}\times \log C$$

In the applied adsorption isotherms, θ represents the portion of the metal surface covered by inhibitor molecules, whereas *K*_ads_ corresponds to the adsorption equilibrium constant. The inhibitor concentration (*C*) is expressed in g/L. It is well established that, in acidic media, many corrosion inhibitors exhibit adsorption behavior consistent with the Langmuir model. Based on the calculated *K*_ads_ values, the standard Gibbs free energy of adsorption (Δ*G*°_ads_) can be determined using eq 5, in which *C* denotes the solvent concentration, assumed to be 1000 g/L for water. Negative values of Δ*G*°_ads_ confirm that the adsorption process occurs spontaneously from a thermodynamic perspective. Typically, |Δ*G*°_ads_| values lower than 20 kJ/mol are characteristic of physisorption, while values greater than 40 kJ/mol indicate chemisorption.^40^ Values falling within the intermediate range of 20–40 kJ/mol are generally interpreted as evidence for the simultaneous contribution of both adsorption mechanisms.

5 $${{\Delta G}^{{}^{\circ}}}_{\mathrm{ads}}=-R\times T\times \ln\left(K_{\mathrm{ads}}\times C\right)$$

The estimation of this thermodynamic parameter is commonly achieved by fitting inhibition efficiency data to adsorption isotherm models, assuming that the inhibition efficiency (IE) directly reflects the surface coverage (θ). However, Walczak, Morales-Gil, and Lindsay^41^ emphasized that the validity of the Langmuir adsorption model depends on strict assumptions, including monolayer adsorption, the attainment of dynamic equilibrium, uniformity of adsorption sites, and the absence of lateral interactions between adsorbed species. In corrosion systems, true dynamic equilibrium is rarely achieved; instead, a steady-state condition is established, as indicated by the stabilization of the open circuit potential (OCP). Moreover, the inherent heterogeneity of corroding metal surfaces challenges the assumption of equivalent adsorption sites. Under these conditions, equating surface coverage with inhibition efficiency may lead to inaccuracies, thereby affecting the reliability of the calculated standard Gibbs free energy of adsorption (Δ*G*°_ads_). In addition, a satisfactory fit to a given isotherm does not necessarily imply that it provides the most appropriate physical description of the system, since inhibition efficiency does not always correspond directly to surface coverage.

Kokalj^42^ further argued that, even when alternative approaches are employed to estimate adsorption free energies, this parameter alone is not a definitive criterion for identifying the adsorption mechanism. This limitation arises because Δ*G*°_ads_ includes entropic contributions; adsorption typically restricts molecular translational and rotational freedom, leading to a decrease in entropy. Consequently, adsorption free energies are generally less exothermic than the corresponding adsorption enthalpies. Additionally, for macromolecular inhibitors, physical adsorption can rival, and even exceed, chemisorption in strength, as long-range van der Waals interactions allow multiple simultaneous weak interactions with the metal surface. Conversely, chemisorption may require structural rearrangements or bond distortions that consume energy, thereby constraining the magnitude of the adsorption enthalpy.

Kokalj^43^ emphasizes that computational methods provide deeper insight into adsorption behavior by enabling detailed examination of molecule–surface bond lengths and the associated electronic structure of the interfacial interactions.

Therefore, in this work, surface coverage was not considered as a measure of similar inhibition efficiency. Instead, valuable information on adsorption will be provided through molecular modeling.

#### 2.2.2 Surface Analysis–Scanning Electron Microscopy (SEM)

SEM–EDS examinations were performed on specimens obtained from gravimetric corrosion tests conducted at 333 K in order to assess surface morphology and the uniformity of the surface layer formed on polished samples exposed to corrosive media with and without the inhibitor. Selected specimens were analyzed in their as-exposed condition, without any post-test cleaning, as illustrated in Figure 6. In this work, the term “cleaned samples” refers to coupons that were mechanically cleaned after the weight-loss measurements, whereas “film” designates samples analyzed directly after immersion, without any cleaning treatment. To ensure representative elemental analysis, at least three different areas on each specimen were examined, allowing the estimation of standard deviations for the elemental compositions reported in Table 2.

**Figure 6:**
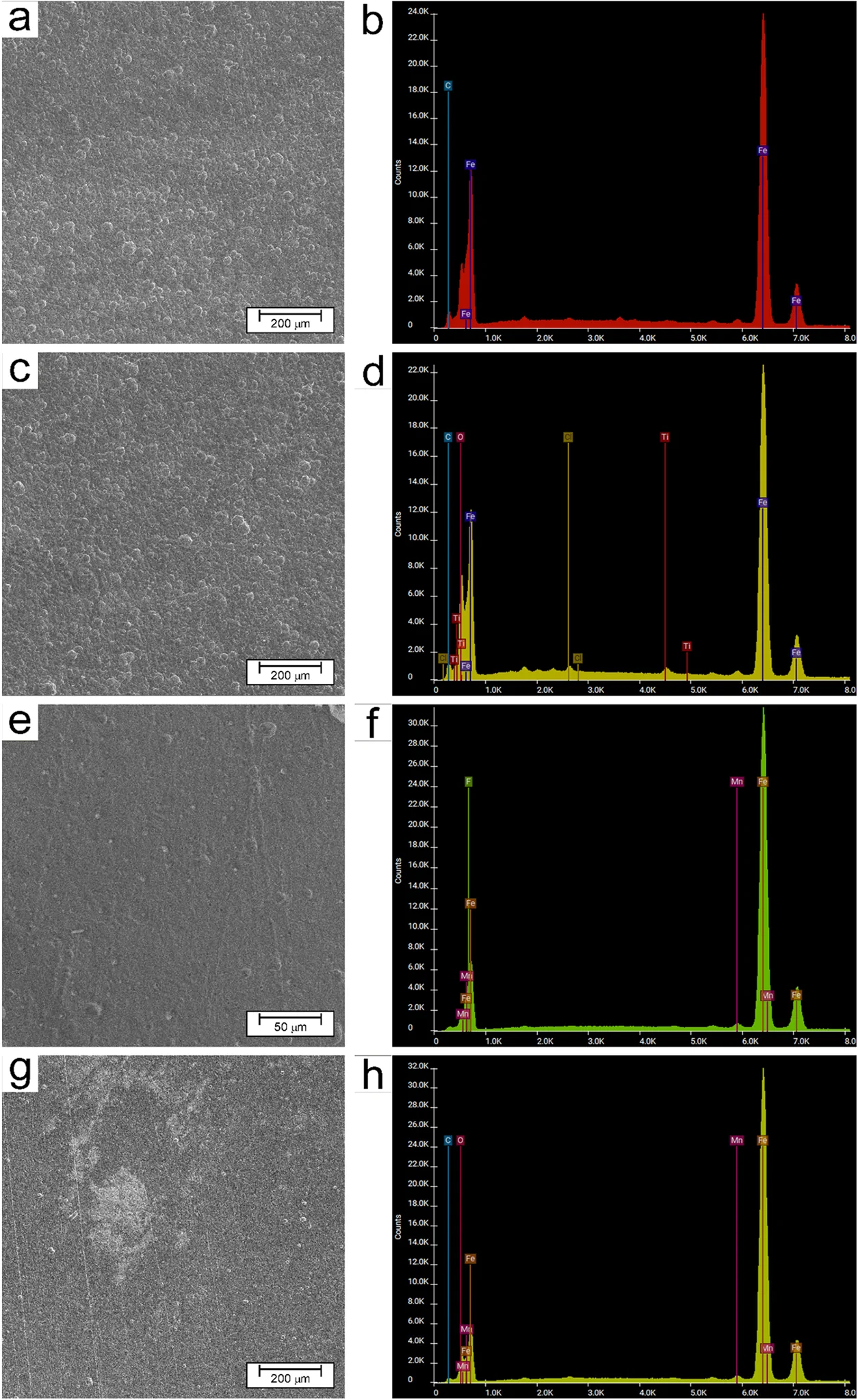
SEM-EDX micrographs of API P110 carbon steel specimens: (a, b) cleaned samples after acidification tests at 333 K without inhibitor; (c, d) “Film” samples after acidification tests at 333 K without inhibitor film; (e, f) cleaned samples after acidification tests at 333 K with 1500 ppm of inhibitor; (g, h) “Film” samples after acidification tests at 333 K with 1500 ppm of inhibitor (film).

**Table 2:** Elemental Composition Identified through SEM–EDX Analysis (Fe, C, O, F, and Mn) for Four Samples (Blank/Cleaned, Blank/Film, 1500 ppm/Cleaned, and 1500 ppm/Film), Mass Content of Each Element Detected (% Mass), and Element Ratios (C/Fe and O/Fe, %)

test	elements	% mass	C/Fe (%)	O/Fe (%)
Blank/Cleaned (a)	Fe	77.2 ± 1.7	29.3	-
	C	22.6 ± 1.5		
Blank/Film (b)	Fe	75.2 ± 10.6	23.3	12.9
	C	17.6 ± 6.9		
	O	9.7 ± 0.5		
1500 ppm/Cleaned (c)	Fe	89.6 ± 9.6	-	-
	F	3.1 ± 0.5		
	Mn	1.7 ± 0.2		
1500 ppm/Film (d)	Fe	81.7 ± 8.4	22.9	11.1
	C	18.8 ± 4.1		
	O	9.1 ± 1.4		
	Mn	1.6 ± 0.2		
	F	3.5 ± 0.8		

Table 2 summarizes the elemental weight percentages obtained from EDS analyses, along with the calculated carbon-to-iron (C/Fe) and oxygen-to-iron (O/Fe) ratios. A comparison between the Inhibitor/Film and Blank/Film conditions reveals similar C/Fe and O/Fe values. Nevertheless, in the presence of the inhibitor, carbon and oxygen are predominantly associated with the organic inhibitory layer, whereas in the uninhibited system, these elements originate mainly from corrosion products. Moreover, SEM micrographs (Figure 6) show that samples exposed to the inhibitor exhibit a smoother and more homogeneous surface with fewer defects (e, g), supporting the formation of a protective film and confirming the inhibitor’s effectiveness.

### 2.3 Electrochemical Tests

#### 2.3.1 Open Circuit Potential (OCP)

Figure 7 illustrates the open-circuit potential (*E*_OCP_) of the working electrode during 2 h of immersion, evaluated under uninhibited conditions and in the presence of 1500 ppm of λ-carrageenan. The measurements were conducted under both static (0 rpm) and controlled hydrodynamic conditions at 500 and 1500 rpm. In all tested conditions, the *E*_OCP_ exhibits an initial transient response followed by convergence toward a quasi-stationary value after approximately 3000 s, reflecting stabilization of the metal/electrolyte interface. At 0 rpm, although λ-carrageenan initially shifts the *E*_OCP_ toward more noble values, after 2 h of immersion, the *E*_OCP_ values of the inhibited and blank systems become nearly identical under the same conditions.

**Figure 7:**
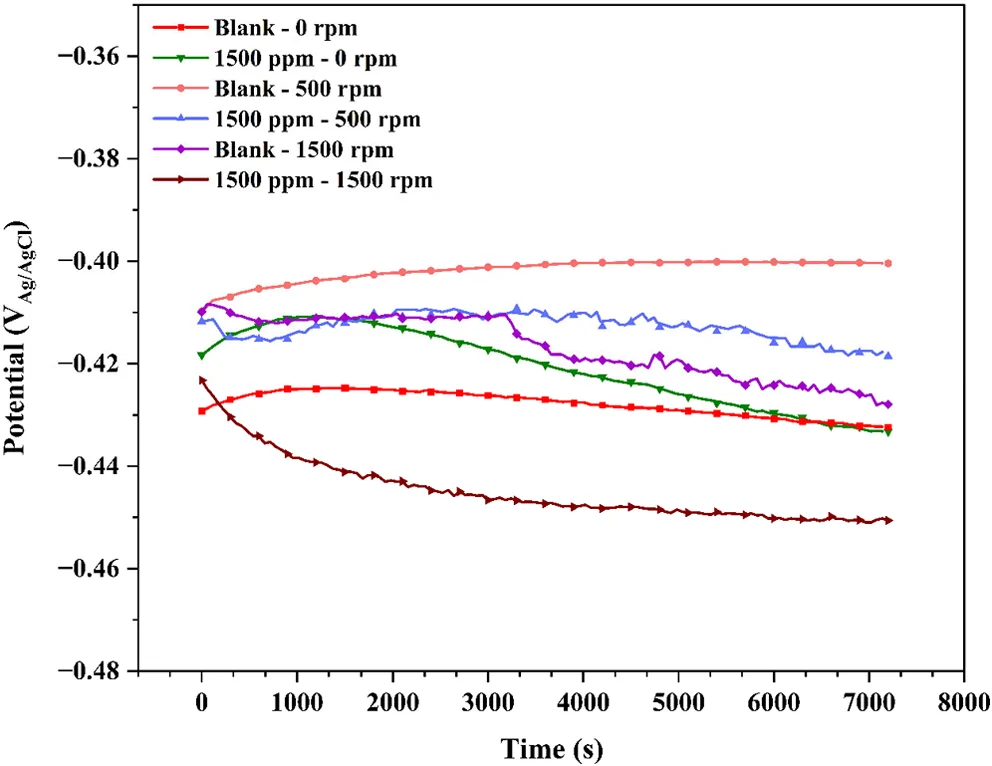
Open circuit potential (OCP) plots (potential in volts versus time in seconds) for API P110 carbon steel in 1 M HCl at 303 K for the blank and 1500 ppm of λ-carrageenan under static (0 rpm) and hydrodynamic conditions (500 and 1500 rpm).

For hydrodynamic conditions, at 500 rpm, both blank and inhibited tests exhibit shifts to nobler potentials. As discussed below, the imposition of hydrodynamic conditions enhances mass transport in the electrolyte, thereby promoting the adsorption of inhibitor molecules, as evidenced in the system containing 1500 ppm of λ-carrageenan. In the absence of inhibitor, the applied rotation speed may instead facilitate the accumulation of corrosion products on the metal surface.^44^ At a higher rotation rate of 1500 rpm, the open-circuit potential of the blank system shifts back toward values comparable to those observed under static conditions, whereas the inhibited system displays a shift toward less noble potentials. This behavior indicates that such high flow intensity may promote partial removal of both inhibitor molecules and surface oxides from the substrate. Moreover, under both static and flow conditions, the relatively small potential difference between the uninhibited and inhibited systems (approximately 85 mV vs Ag/AgCl) suggests that the inhibitor acts through a mixed-type adsorption mechanism.

#### 2.3.2 Linear Polarization Resistance (LPR)

LPR measurements were employed to monitor the temporal evolution of the corrosion rate during 24 h of immersion. The measurements were performed under the condition that exhibited one of the highest inhibition efficiencies in the weight loss measurements (1500 ppm, 303 K), with data acquired at 4 h intervals. Figure 8 shows that the corrosion rate increased with immersion time in both the blank and inhibited systems, with the uninhibited solution exhibiting a corrosion rate approximately 306% higher than that of the inhibited solution at the final measurement. The observed increase, even with λ-carrageenan, suggests that the biopolymer is desorbing from the metal surface, exposing the metal to the acidic environment and, consequently, increasing the corrosion rate over the analysis time.

**Figure 8:**
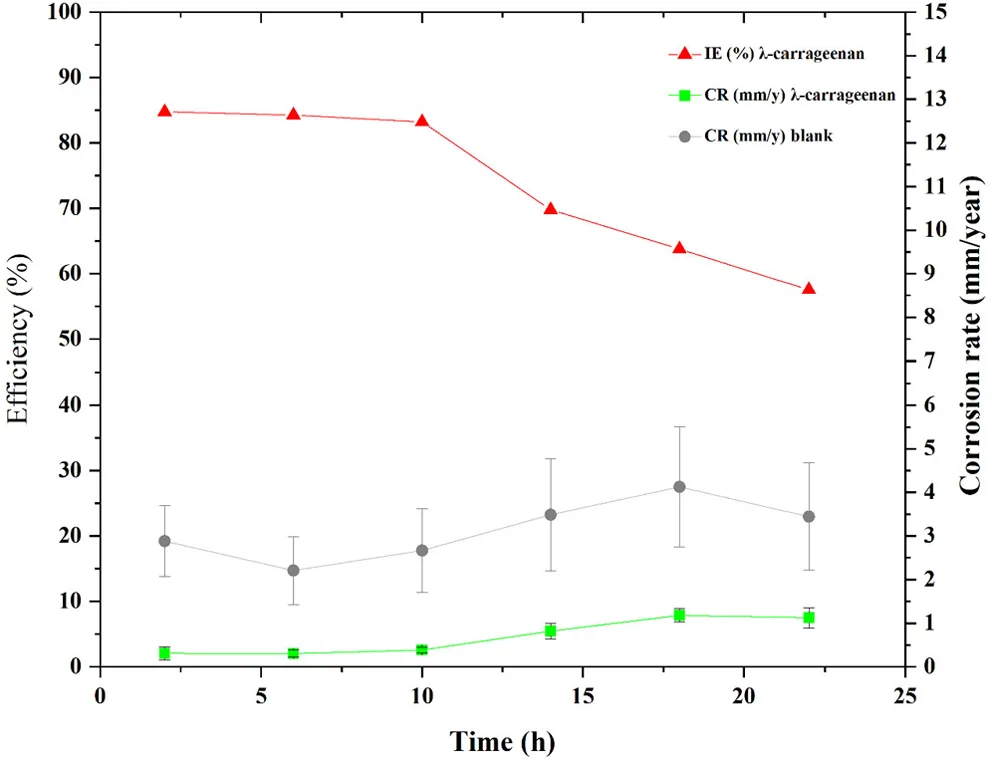
Real-time corrosion rate (mm/year) and efficiency (%) plot for carbon steel in 1 M HCl at 303 K for λ-carrageenan (1500 ppm) and the blank, under static condition (0 rpm).

The influence of hydrodynamic conditions was evaluated at 303 K using rotation speeds of 500 and 1500 rpm. The selected rotational speeds fall within ranges commonly reported for organic corrosion inhibitors.^44–49^

Ashassi-Sorkhabi and Asghari^45^ examined the inhibition behavior of l-methionine in 1 M H_2_SO_4_ under rotation rates from 0 to 2200 rpm, reporting inhibition efficiencies between 50.0% and 60.5% based on potentiodynamic polarization measurements. Solomon et al.^46^ investigated an imidazoline inhibitor for low-carbon steel in 15% HCl at 0, 500, 1000, and 1500 rpm. In that study, the polarization resistance increased from 75.99 Ω·cm^2^ at static conditions to 190.90 and 198.10 Ω·cm^2^ at 500 and 1000 rpm, respectively, followed by a sharp decrease to 24.74 Ω·cm^2^ at 1500 rpm.

Odewunmi et al.^44^ evaluated a dicationic monomer (NDTHDC) and its polymeric form (poly-NDTHDC) on API X60 carbon steel in 15 wt % HCl under flow conditions of 500, 1000, and 1500 rpm. Their results showed enhanced inhibition performance under moderate hydrodynamic conditions, with efficiencies increasing from 89.2% and 90.2% at 0 rpm to 90.9% and 91.6% at 1000 rpm for NDTHDC and poly-NDTHDC, respectively, followed by a decline at 1500 rpm. Similarly, Mu’azu et al.^49^ studied a cationic gemini surfactant on low-carbon steel in 10 wt % H_2_SO_4_ and 15 wt % HCl over a range of 0–1600 rpm at 298 K, observing a decrease in inhibition efficiency from 95.5% under static conditions to 87.9% at the highest rotation speed.

For this work, the inhibition efficiency and polarization resistance values at 500 and 1500 rpm are summarized in Table 3. The hydrodynamic regime was assessed through the Reynolds number using eq 6, where *r*, ω, and *k*_v_ correspond to the rotating disk radius (mm), angular velocity (rad/s), and kinematic viscosity (mm^2^/s), respectively. From Reynolds numbers in Table 3, all experiments were under laminar flow conditions.

6 Re=r2·ωkv<105for laminar flow

**Table 3:** Linear Polarization Resistance (LPR) Data (*R*_p_, Ω cm^2^; IE_LPR_, %), Kinematic Viscosity (mm^2^/s), and Reynolds Numbers for API P110 Carbon Steel in 1 M HCl in the Absence and Presence of 1500 ppm of λ-Carrageenan at 0, 500, and 1500 rpm

rotation speed (rpm)	inhibitor concentration (ppm)	kinematic viscosity (mm^2^/s)	reynolds	*R*_p_ (Ω·cm^2^)	IE_LPR_ (%)
0	Blank	0.997	-	41.1 ± 4.2	-
	1500	1.445	-	269.8 ± 83.3	84.7
500	Blank	0.997	1299.6	19.8 ± 3.6	-
	1500	1.445	896.8	195.1 ± 21.3	89.8
1500	Blank	0.997	3898.7	21.9 ± 8.5	-
	1500	1.445	2690.5	164.5 ± 12.2	86.6

A moderate increase in inhibition efficiency was observed when the rotation speed was raised from static conditions to 500 rpm, with efficiency rising from 84.7% to 89.8%. At 1500 rpm, the inhibition efficiency remained high (86.6%), showing only a slight difference relative to that obtained at 500 rpm. Considering the relatively small difference in inhibition efficiency together with the experimental scatter associated with the polarization resistance measurements, particularly for the inhibited condition, the inhibition efficiencies obtained at 500 and 1500 rpm may be regarded as comparable. These results indicate that hydrodynamic conditions favor the inhibition performance of λ-carrageenan. Enhanced mass transport under flow is expected to facilitate the transport of inhibitor molecules to the metal surface, contributing to the formation and maintenance of a protective adsorbed layer. Within the investigated hydrodynamic conditions, λ-carrageenan maintained high inhibition efficiency, indicating that fluid flow did not adversely affect its protective performance.

#### 2.3.3 Potentiodynamic Polarization (PDP)

Figure 9 presents the potentiodynamic polarization responses of carbon steel in uninhibited and inhibited acidic media, comparing the blank solution with the system containing 1500 ppm of λ-carrageenan, at 0, 500, and 1500 rpm. The corrosion current densities (*i*_corr_) and corrosion potential (*E*_corr_) obtained from Tafel extrapolation are summarized in Table 4. In the presence of the inhibitor, the polarization curves slightly displace toward more cathodic potentials. Nevertheless, the shift in corrosion potential (*E*_corr_) relative to the blank remains lower than 85 mV vs Ag/AgCl, indicating that λ-carrageenan behaves as a mixed-type inhibitor, affecting both the anodic iron dissolution and the cathodic hydrogen evolution reactions.^22,50^

**Figure 9:**
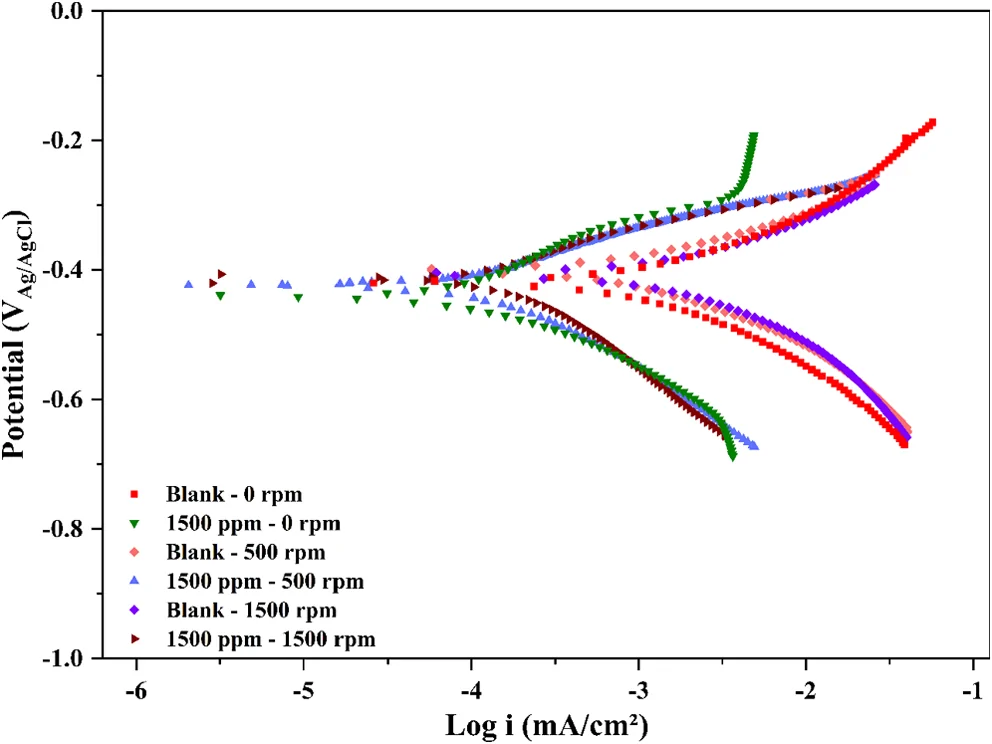
Anodic and cathodic polarization curves for carbon steel in 1 M HCl at 303 K for the blank and λ-carrageenan (1500 ppm), under static (0 rpm) and hydrodynamic conditions (500 and 1500 rpm), after 2 h of immersion.

**Table 4:** Potentiodynamic Polarization (PDP) Data (*E*_corr_, mV; *i*_*c*orr_, mA/cm^2^; β_anodic_, mV/dec; β_cathodic_, mV/dec; IE, %) for the Blank and λ-Carrageenan (1500 ppm) at 303 K in 1 M HCl, after 2 h of Immersion, under Static (0 rpm) and Hydrodynamic Conditions (500 and 1500 rpm)

Rotation Speed (rpm)	Test	*E*_corr_ (mV)	*i*_corr_ (mA/cm^2^)	β_a_ (mV/dec)	β_c_ (mV/dec)	Corrosion Rate (mm/year)	IE (%)
0	Blank	–429	0.36 ± 0.15	64	66	4.3 ± 1.8	-
	1500 ppm	–450	0.06 ± 0.03	56	29	0.8 ± 0.4	81.5
500	Blank	–399	0.34 ± 0.12	31	36	4.0 ± 1.4	-
	1500 ppm	–432	0.05 ± 0.01	63	58	0.6 ± 0.1	84.7
1500	Blank	–393	0.54 ± 0.23	51	57	6.2 ± 2.7	-
	1500 ppm	–445	0.05 ± 0.03	55	53	0.5 ± 0.3	92.1

The modification of the anodic branch shape and the noticeable decrease in anodic current densities upon addition of λ-carrageenan indicate a reduction in the rate of iron dissolution in the acidic environment.^51,52^ Moderate changes were also observed in the anodic (β_a_) and cathodic (β_c_) Tafel slopes after inhibitor addition. However, no systematic variation indicating a preferential modification of either the anodic or cathodic reaction mechanism was observed under static and hydrodynamic conditions. Instead, the combined reduction in corrosion current density and the small displacement of *E*_corr_ suggest that λ-carrageenan inhibits corrosion predominantly through adsorption and blocking of active surface sites, reducing the kinetics of both anodic and cathodic reactions without evidence of a significant change in the electrochemical reaction mechanisms, which is consistent with the behavior of a mixed-type inhibitor.^53^

Moreover, the corrosion current densities measured in the inhibited system are markedly lower than those obtained for the blank solution, reflecting an overall decrease in corrosion rate. Based on these results, the inhibition efficiency was calculated to be 81.5% under static conditions, increasing to approximately 84.7% at 500 rpm and reaching 92.1% at 1500 rpm, demonstrating the enhanced protective effect of λ-carrageenan under hydrodynamic conditions.

### 2.4 Molecular Modeling

#### 2.4.1 DFT Results

Density functional theory (DFT) calculations were employed in the gas phase to examine the structural, electronic, and quantum characteristics of λ-carrageenan models comprising one, four, and eight repeating units. The models were considered in their fully protonated SO_3_H forms and optimized, as illustrated in Figure 10. The optimized monomeric structure exhibits an almost planar geometry, which can be attributed to strong intramolecular hydrogen bonding between sulfonic and hydroxyl functionalities. In contrast, the oligomeric models containing four and eight monomer units, while also stabilized by extensive hydrogen-bonding interactions, display a pronounced tendency to adopt more compact, folded, and helical-like conformations. Given that oxygen atoms are recognized as favorable adsorption centers for interaction with metallic substrates, the abundance of OH and SO_3_H groups within the λ-carrageenan framework is expected to facilitate adsorption through multiple binding sites. In particular, the sulfonic groups, which experience lower steric constraints, are especially well positioned to interact effectively with the metal surface.

**Figure 10:**
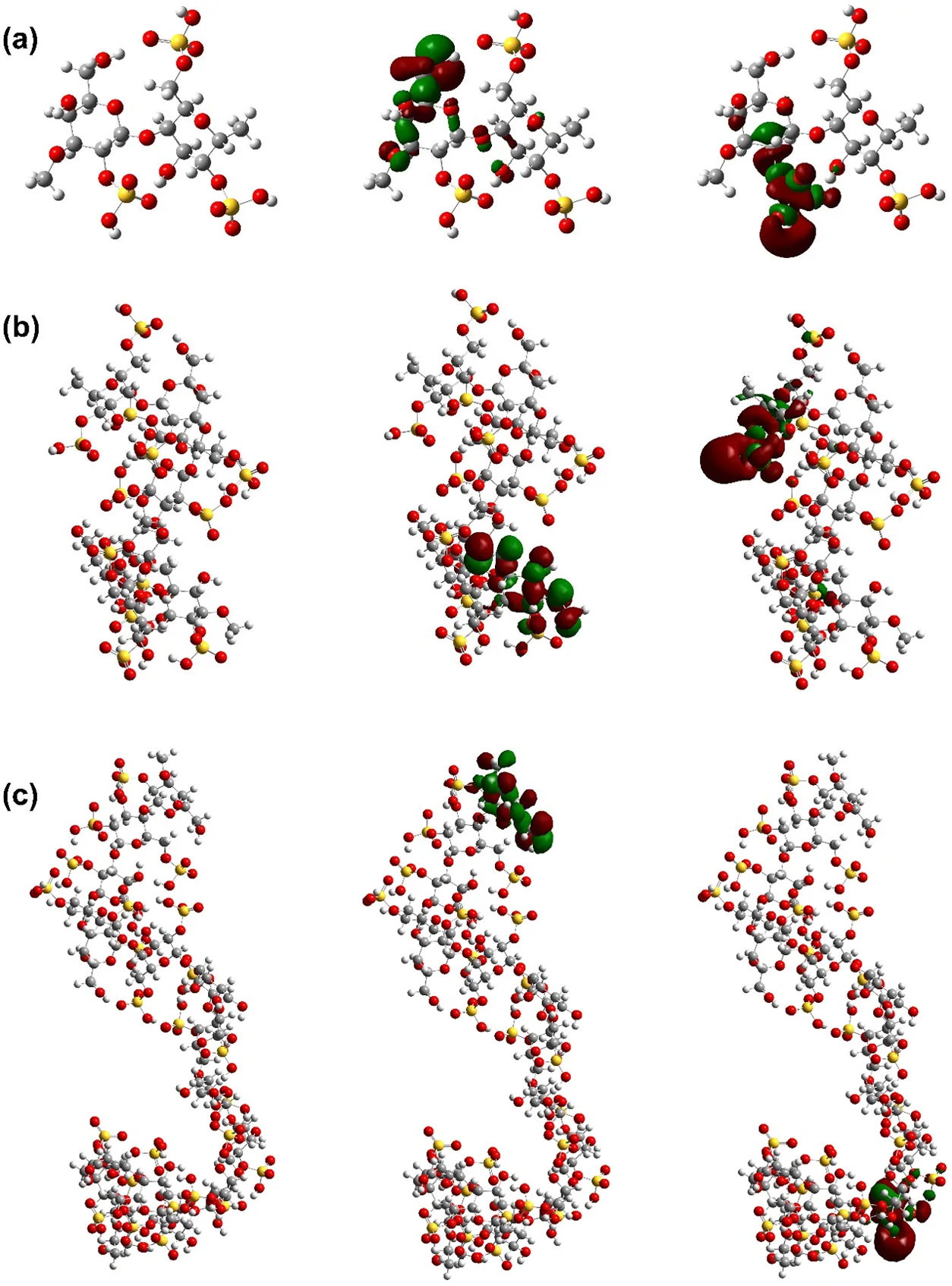
(a) Optimized geometries of λ-carrageenan models containing 1, 4, and 8 monomeric units in their neutral SO_3_H form; (b) distribution of the highest occupied molecular orbital (HOMO); and (c) distribution of the lowest unoccupied molecular orbital (LUMO). Atom color scheme: red = oxygen, yellow = sulfur, gray = carbon, and white = hydrogen.

The electron density distribution of λ-carrageenan exhibits comparable features for all three molecular models considered, as shown in Figure 10. In each case, the highest occupied molecular orbital (HOMO) is mainly localized on the oxygen atoms of hydroxyl and ether functionalities, identifying these sites as the primary centers for electron donation toward the metal surface. This behavior is supported by electron density maps, which reveal enhanced charge accumulation on hydroxyl groups, followed by the oxygen atoms of cyclic ethers and bridging ether linkages connecting the six-membered rings.

In contrast, the lowest unoccupied molecular orbital (LUMO) is predominantly associated with the sulfonic groups, consistent with their comparatively lower electron density. This localization suggests that the SO_3_H groups act as efficient electron-accepting sites, where positive charge can be stabilized through resonance within the sulfonate structure. The coexistence of electron-donating centers (hydroxyl and ether oxygens) and electron-accepting regions (sulfonic groups) points to a cooperative electronic mechanism that enhances the adsorption of λ-carrageenan onto metallic surfaces in corrosive environments.

The quantum parameters, such as the energies of the highest occupied molecular orbital (*E*_HOMO_) and the lowest vacant molecular orbital (*E*_LUMO_), the energy gap (Δ*E*) between *E*_HOMO_ and *E*_LUMO_, dipole moment (μ), the global electronegativity (χ), the hardness (η) and the softness (σ), were obtained using the optimized structure corresponding data. From the calculated HOMO and LUMO values, it is possible to determine, through the mathematical expressions described in eqs 7–9, the global electronegativity (χ), chemical hardness (η), and softness (σ).

7 $$\chi =-\frac{E_{\mathrm{HOMO}}+E_{\mathrm{LUMO}}}{2}$$

8 $$\eta =\frac{{E_{\mathrm{LUMO}}+E}_{\mathrm{HOMO}}}{2}$$

9 $$\sigma =\frac{1}{\eta }$$

The computed quantum descriptors of the λ-carrageenan models are summarized in Table 5. All neutral λ-carrageenan configurations displayed quantum characteristics comparable to other reported organic inhibitors.^35,54–59^ In the neutral monomeric model, the energy gap (Δ*E* = 7.05 eV) and a LUMO value near zero (−0.03 eV) indicate moderate molecular stability and limited electron-accepting ability. As the chain length increased to four and eight repeating units, the energy gap narrowed (Δ*E* = 6.36 and 6.28 eV, respectively), while the dipole moment rose significantly (μ = 20.57 and 37.60 D). These features reveal enhanced molecular reactivity and a stronger tendency toward surface polarization, facilitating adsorption through dipole–metal interactions.

**Table 5:** Quantum Parameters of λ-Carrageenan (in Its Ionic and Neutral Forms, with 1, 4, and 8 Monomeric Units) and Other Organic Molecules from the Literature in the Neutral State

quantum properties	*E*_HOMO_ (eV)	*E*_LUMO_ (eV)	Δ*E* (eV)	μ (D)	χ (eV)	η (eV)	σ (e/V)
Ionic λ-carrageenan (monomer)	+2.57	+6.74	4.17	3.64	–4.66	2.09	0.48
Neutral λ-carrageenan (monomer)	–7.08	–0.03	7.05	3.64	3.56	3.53	0.28
Neutral λ-carrageenan (tetramer, 4 repeating units)	–6.92	–0.56	6.36	20.57	3.74	3.18	0.314
Neutral λ-carrageenan (octamer, 8 repeating units)	–6.93	–0.65	6.28	37.60	3.79	3.14	0.318
*Peumusboldus* ^55^	–5.28	–0.52	4.76	2.33	2.90	2.38	0.42
Cellulose–pectin–TiO_2_ ^58^	–8.00	–1.25	6.75	-	-	-	-
Arabinogalactan^59^	–6.94	–0.55	6.40	6.96	3.75	3.20	-

In contrast, the ionic λ-carrageenan model (3 × SO_3_^–^) exhibited a positive HOMO energy (+2.57 eV) and a smaller bandgap (4.17 eV), confirming its higher reactivity and superior ability to exchange charge with metallic surfaces under acidic conditions. These findings are consistent with its role as a polyanionic species, capable of coordinating with metal cations while maintaining structural integrity. Overall, the combination of high dipole moments, reduced energy gaps, and favorable frontier orbital distributions indicates that λ-carrageenan exhibits strong electronic properties for corrosion inhibition, both in its neutral and ionic forms.

In this work, λ-carrageenan was examined in acidic environments to understand how protonation affects its molecular stability and electronic properties. The polymer presents multiple potential protonation sites associated with oxygen and sulfur atoms, and its acidity was quantified through the protonation energy (Δ*E*_prot_), obtained from the energy difference between the protonated (λ-carrageenan + H^+^) and neutral (λ-carrageenan) forms, as expressed in eq 10. The calculated protonation energies are summarized in Table 6.

10 $${\Delta E}_{\mathrm{Prot}}={\Delta E}_{\mathrm{inhibitor}+}-{\Delta E}_{\mathrm{inhibitor}}$$

**Table 6:** Quantum Parameters of Protonated Neutral λ-Carrageenan (1 Monomer) at Different Protonation Sites (Heteroatoms) in the Gas Phase

quantum properties	*E*_HOMO_ (eV)	*E*_LUMO_ (eV)	Δ*E* (eV)	μ (D)	χ (eV)	η (eV)	σ (eV^–1^)	Δ*E*_prot_ (kcal/mol)
λ-carrageenan (SO_3_H)—O(10)	–9.49	–5.08	4.41	12.46	7.29	2.20	0.45	–211.04
λ-carrageenan (SO_3_H)—O(12)	–9.59	–4.80	4.79	14.18	7.20	2.40	0.42	–222.46
λ-carrageenan (SO_3_H)—O(14)	–9.72	–6.13	3.60	6.91	7.93	1.80	0.56	–217.98
λ-carrageenan (SO_3_H)—O(16)	–9.99	–4.55	5.43	6.29	7.27	2.72	0.37	–217.52
λ-carrageenan (SO_3_H)—O(27)	–9.31	–4.78	4.53	6.30	7.05	2.27	0.44	–220.84
λ-carrageenan (SO_3_H)—O(29)	–8.96	–5.79	3.17	7.05	7.37	1.59	0.63	–220.84
λ-carrageenan (SO_3_H)—O(30)	–9.32	–4.59	4.72	7.37	6.96	2.36	0.42	–215.73
λ-carrageenan (SO_3_H)—O(43)	–9.49	–6.29	3.20	7.89	7.89	1.60	0.63	–215.37
λ-carrageenan (SO_3_H)—O(55)	–9.39	–5.78	3.60	7.59	7.59	1.80	0.56	–220.38

The protonation of the molecule was investigated to evaluate its structural stability and electronic behavior under acidic conditions. After protonation, all hydrogen atoms remained covalently bonded to the sulfonic oxygen atoms, and no bond rupture or molecular distortion was observed. This finding demonstrates that the polymeric framework can accommodate additional positive charge without structural degradation.

The electronic parameters calculated for the protonated λ-carrageenan models reveal enhanced molecular reactivity relative to the neutral species. The increase in *E*_HOMO_ values reflects a greater propensity of the protonated form to donate electrons to the metal surface, thereby promoting adsorption through electrostatic interactions and donor–acceptor bonding. At the same time, a moderate increase in *E*_LUMO_ energy suggests improved ability to accept electronic density from the iron atoms, reinforcing bidirectional charge transfer at the interface. In addition, the dipole moment also rises slightly upon protonation, reflecting stronger molecular polarization and greater affinity for the charged metal surface.

#### 2.4.2 Molecular Dynamics Results

During the equilibration stage of 20 ns at 303 K, the λ-carrageenan monomer remained adsorbed on the metallic surface at an average distance of approximately 2.7 Å. The polymer interacted effectively with the 1 M HCl medium while maintaining a stable configuration near the Fe surface. The system exhibited a homogeneous ion distribution, with no chloride or hydronium aggregation, indicating that the molecular-dynamics setup accurately reproduced the bulk electrolyte behavior.

In the subsequent heating ramp, a continuous 150 ns NVT simulation was performed, in which the temperature was progressively increased in three fixed stages: the first 50 ns at 303 K, followed by a temperature increase from 303 to 318 K from 50 to 100 ns, and finally to 333 K from 100 to 150 ns. Across the entire temperature range, λ-carrageenan exhibited robust and sustained interactions with the iron surface, with no evidence of desorption or major structural rearrangement of the adsorbed layer. This adsorption stability is largely attributed to the abundance of heteroatoms, particularly oxygen and sulfur, distributed along the biopolymer chain. These atoms create multiple attachment sites, primarily through hydroxyl and sulfonic functionalities, which can establish hydrogen bonding and electrostatic interactions with surface hydroxyl groups or exposed iron centers, as illustrated in Figure 11.

**Figure 11:**
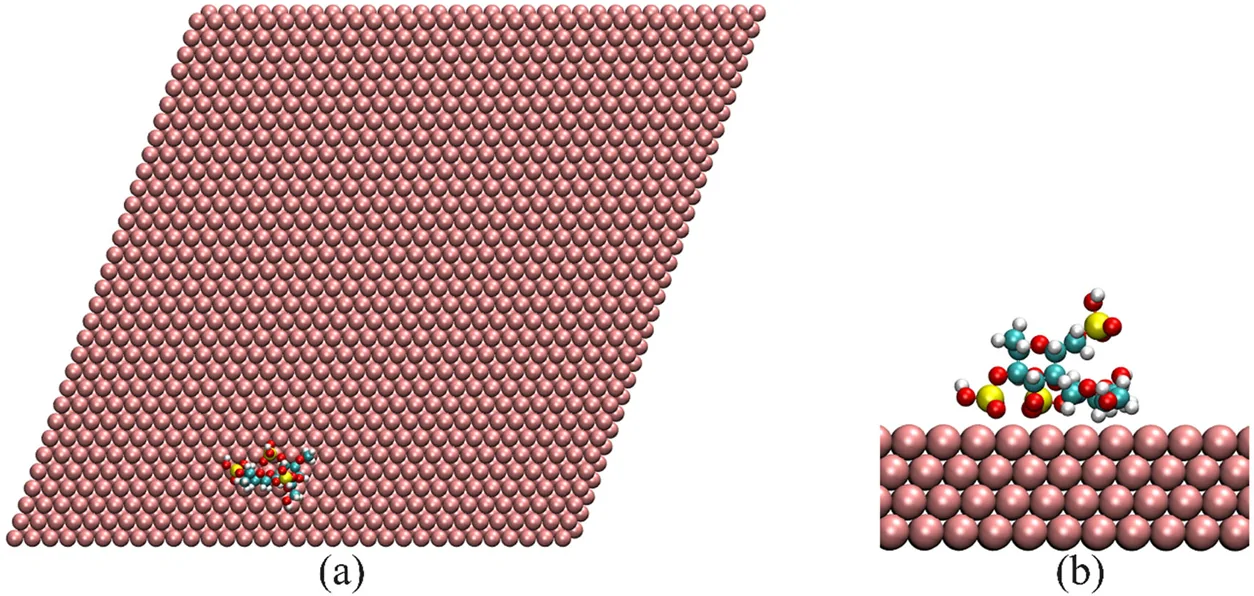
Top (a) and side (b) views of the interaction between λ-carrageenan and the Fe surface at 30 °C. Water (H_2_O), hydronium (H_3_O^+^), and chloride (Cl^–^) molecules were omitted for clarity. Atom color scheme: iron = pink, carbon = cyan, oxygen = red, sulfur = yellow, and hydrogen = white.

The adsorption energy (*E*_int_) was calculated to quantify the interaction strength between λ-carrageenan and the metallic surface according to eq 11

11 $$E_{\mathrm{int}}=E_{\mathrm{total}}-\left(E_{\mathrm{surface}+\mathrm{solution}}+E_{\mathrm{polymer}}\right)$$

where *E*_total_ corresponds to the total energy of the complete system composed of the iron surface, the 1 M HCl, and the λ-carrageenan molecule; *E*_surface+solution_ is the energy of the metallic surface in contact with 1 M HCl electrolyte without the polymer; and *E*_polymer_ represents the energy of the isolated λ-carrageenan monomer.

In each temperature stage (303, 318, and 333 K), the average total energy of the last 10 ns of simulation was used to ensure convergence and minimize instantaneous fluctuations. The resulting adsorption energies were −158.3, −176.4, and −179.3 kcal/mol, respectively. The negative values confirm a spontaneous and strongly exergonic adsorption process, while the gradual increase in magnitude (Δ ≈ −21 kcal/mol between 303 and 333 K) indicates that the polymer–surface interaction becomes more favorable with increasing temperature. The most pronounced stabilization occurs between 303 and 333 K. In contrast, from 318 to 333 K, the change in adsorption energy is relatively small, indicating that the system has reached a near-saturation regime where most of the polymer’s active sites are already engaged with the metallic surface. The slight additional stabilization at 333 K may result from subtle conformational rearrangements and partial dehydration of the interfacial region, which enhances local packing and strengthens van der Waals and electrostatic interactions. Moreover, the higher temperature itself may start to influence the adsorption dynamics, reducing the lifetime of weaker hydrogen bonds while promoting faster reorganization of solvent molecules and counterions near the surface. Despite this thermal perturbation, the overall adsorption remains exergonic and robust, indicating that enthalpic contributions continue to dominate over entropic effects and that λ-carrageenan retains its strong affinity for the metallic substrate even under elevated thermal conditions.

The outcomes of the molecular dynamics simulations are in good agreement with the experimental corrosion results. Gravimetric tests revealed a decline in inhibition efficiency at elevated temperatures and extended immersion periods, which is attributed to partial desorption of the protective layer, a characteristic behavior of inhibition systems dominated by physical adsorption. In the simulations, the modest increase in adsorption energy between 318 and 333 K mirrors this thermodynamic trend: while moderate temperature elevation enhances molecular mobility and promotes adsorption, further heating introduces thermal fluctuations that progressively weaken hydrogen-bond interactions. Consequently, the molecular-level findings explain the experimental trend, confirming that the adsorption of λ-carrageenan is primarily governed by strong yet temperature-sensitive physical interactions that allow the formation of a stable protective film under moderate thermal conditions.

From the molecular modeling perspective, λ-carrageenan also exhibits structural and electronic features that favor adsorption on the iron surface. DFT calculations indicate that hydroxyl and ether oxygen atoms are the main electron-donating sites, whereas the sulfonic groups behave as electron-accepting regions, suggesting a possible donor–acceptor contribution to the interfacial interaction. At the same time, molecular dynamics simulations revealed spontaneous and stable adsorption, with strongly negative adsorption energies and the formation of a protective layer sustained by multisite interactions involving mainly SO_3_H and OH groups. Overall, these results indicate that both physical and chemical contributions may participate in the adsorption process; however, the dominant contribution is physical, especially through electrostatic, van der Waals, and hydrogen-bonding interactions.

## 3 Mechanism of Inhibition

Understanding the inhibition mechanism of λ-carrageenan on carbon steel in 1 M HCl requires a detailed analysis of its molecular structure, electronic properties, and adsorption behavior. Recent studies on biopolymer corrosion inhibitors have highlighted that inhibition can arise from chemisorption, physisorption, or a mixed mechanism, depending on the functional groups and the metal/electrolyte interface.^60–62^ For example, chitosan derivatives containing heteroatoms adsorb on iron surfaces via covalent bond formation.^60^ Similarly, dicarboxylated inulin acts as a Lewis base, donating electron pairs from – COO^–^ groups to vacant d-orbitals of Fe, thereby forming a protective monolayer.^61^ In the case of xylan in H_2_SO_4_, both protonation of the biopolymer and electron transfer from oxygen lone pairs to metal orbitals contribute to a predominantly chemisorption mechanism.^62^

Theoretical calculations show that λ-carrageenan contains several oxygen-containing groups capable of interacting with the metal interface. In mechanistic terms, these groups provide multiple anchoring points along the polymer chain, favoring adsorption through electrostatic attraction, hydrogen bonding, and van der Waals interactions. The donor–acceptor distribution revealed by the electronic-structure analysis suggests that localized chemical interactions may also occur, but their role appears to be complementary rather than dominant. Thus, the inhibition mechanism is better described as the adsorption of a flexible biopolymer through cooperative interfacial contacts distributed over its structure.

Once adsorbed, λ-carrageenan forms a compact barrier layer that hinders the access of aggressive species such as H_3_O^+^, Cl^–^, and H_2_O to the carbon steel surface. This barrier effect is consistent with the lower corrosion current density and higher polarization resistance observed experimentally, as well as with the persistence of inhibition under hydrodynamic conditions. Therefore, the main role of λ-carrageenan is not simply to interact locally with isolated surface sites, but to build a protective interfacial film sustained by multiple simultaneous contacts with the metallic substrate. This adsorption mode is illustrated in Figure 12. Figure 12(a) shows representative interfacial distances involving hydrogen atoms bonded to carbon-containing moieties of the biopolymer, whereas Figure 12(b) shows representative interfacial distances associated with the oxygen-containing groups of the λ-carrageenan structure. Together, these configurations reinforce the multisite nature of adsorption and support the formation of a stable protective layer on the iron surface.

**Figure 12:**
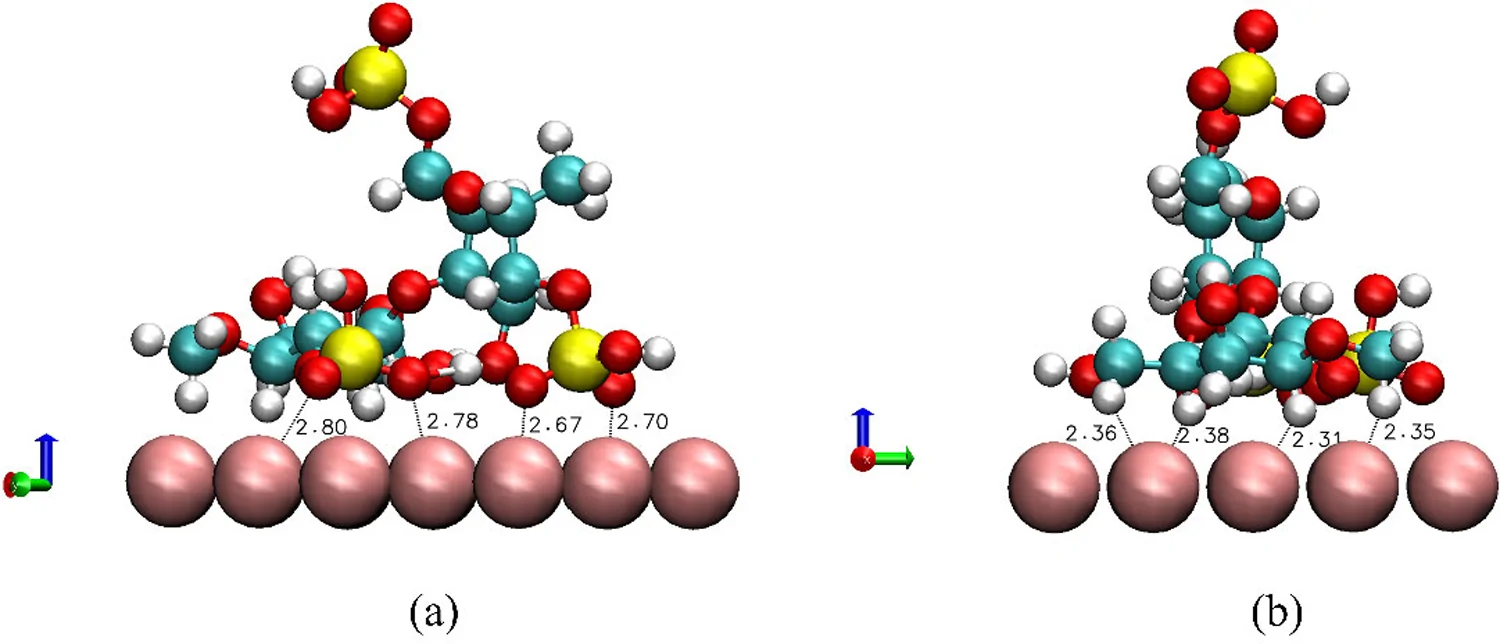
Representative adsorption configurations of λ-carrageenan on the iron surface: (a) interfacial distances involving hydrogen atoms bonded to carbon atoms; (b) interfacial distances involving oxygen-containing groups. Atom color scheme: iron = pink, carbon = cyan, oxygen = red, sulfur = yellow, and hydrogen = white.

## 4 Conclusions

The inhibitory behavior of λ-carrageenan toward carbon steel was systematically assessed in 1 M HCl under both static and flowing conditions. The FTIR analysis confirmed the expected functional groups of λ-carrageenan, including O–H, aliphatic C–H, C=O, and sulfate-related bands, indicating the presence of oxygen and sulfur as potential adsorption sites.

Gravimetric tests revealed that λ-carrageenan acts as an effective corrosion inhibitor for carbon steel in an acidic medium, with inhibition efficiency reaching a saturation concentration of 1000 ppm after 24 h of immersion, beyond which no further improvement is observed due to limited biopolymer solubility. At ambient temperature (303 K), longer exposure times (up to 24 h) favor more effective surface coverage and higher efficiency, attributed to the macromolecular nature of the polysaccharide, which requires extended contact time to achieve complete surface coverage at lower temperatures. Increasing temperature generally raises the corrosion rate and promotes partial desorption, indicating reduced stability of the adsorbed layer. However, when temperature and time are analyzed together, a greater decline in efficiency with increasing temperature is observed at 24 h than at 6 or 15 h of immersion. For instance, at 1000 ppm, efficiency drops by approximately 20% between 303 and 333 K after 24 h, whereas only 9–10% decreases are seen after shorter immersion periods. This pronounced efficiency loss at prolonged exposure under thermal stress suggests structural rearrangement, partial decomposition, or desorption of the biopolymer from the metal surface. Overall, λ-carrageenan offers good protection at moderate temperatures and concentrations, but its performance is limited under prolonged acidic conditions at elevated temperatures, particularly at lower inhibitor concentrations. Surface examination by SEM further confirmed the protective role of λ-carrageenan, revealing a smoother steel surface with fewer defects compared to the uninhibited condition. In addition, future studies using XPS and Raman could complement the proposed mechanism.

Electrochemical tests demonstrated that λ-carrageenan acts as a mixed-type inhibitor of carbon steel in an acidic medium. Linear polarization resistance measurements over 24 h at 1500 ppm and 303 K revealed that, although the corrosion rate increased with immersion time in both uninhibited and inhibited systems, it remained approximately 306% higher in the absence of the biopolymer, indicating sustained, albeit gradually decreasing, protection throughout the immersion period. Under hydrodynamic conditions, both linear polarization resistance and potentiodynamic polarization measurements demonstrated high inhibition performance, confirming that λ-carrageenan remained an effective corrosion inhibitor under the investigated hydrodynamic conditions.

Experimental observations are strongly supported by theoretical investigations. Density Functional Theory calculations revealed that the highest occupied molecular orbital of λ-carrageenan is mainly distributed over the oxygen atoms of hydroxyl and ether groups, whereas the lowest unoccupied molecular orbital is predominantly associated with sulfonic functionalities. This electronic arrangement highlights the coexistence of electron-donating and electron-accepting sites, enabling cooperative interactions with the metal surface. The calculations also revealed that the monomeric unit of λ-carrageenan adopts an almost planar geometry, stabilized by strong intramolecular hydrogen bonding between sulfonic and hydroxyl groups. In contrast, oligomeric models containing four and eight monomer units exhibit a pronounced tendency to adopt more compact, folded, and helical-like conformations, also stabilized by extensive hydrogen-bonding interactions. The abundance of OH and SO_3_H functional groups throughout the polymer framework provides multiple oxygen atoms, which are recognized as favorable adsorption centers for interaction with metallic substrates.

Moreover, DFT results indicated that protonated λ-carrageenan maintains structural stability upon proton addition, without significant conformational distortion, owing to effective charge delocalization across sulfate and hydroxyl functionalities. The protonated forms also exhibit high dipole moments, reduced energy gaps, and favorable frontier orbital distributions, indicating enhanced electronic reactivity and strengthening their interaction with iron.

Classical molecular dynamics corroborated the strong affinity of λ-carrageenan for the iron surface. Throughout the 150 ns simulation period, the polymer remained firmly adsorbed on the iron surface, with adsorption stabilized by multiple interfacial contacts involving mainly SO_3_H and OH groups, as well as additional C–H···surface interactions that contribute to the overall stability of the protective layer. The increase in adsorption energy magnitude with temperature (Δ ≈ −21 kcal/mol between 303 and 333 K) indicates that polymer–surface interactions become more favorable as temperature rises, reaching a near-saturation regime beyond 318 K where most active sites are already engaged with the metal surface. These computational findings align well with experimental gravimetric results: while moderate temperature elevation enhances molecular mobility and promotes adsorption, further thermal stress introduces fluctuations that progressively weaken hydrogen-bond interactions. Thus, λ-carrageenan adsorption is primarily governed by strong yet temperature-sensitive physical interactions, allowing stable protective film formation under moderate thermal conditions, consistent with the observed decline in inhibition efficiency at elevated temperatures due to partial desorption.

## 5 Experimental Section

### 5.1 Solutions Preparation

Figure 13 illustrates the λ-carrageenan used in this study, obtained from Ingredientes Online and the other five types of carrageenans. The biopolymer was first solubilized in distilled water under continuous magnetic stirring at 298 K until a uniform solution was obtained. Subsequently, a series of test solutions was prepared, resulting in concentrations ranging from 0 to 1500 ppm in 1 M HCl.

**Figure 13:**
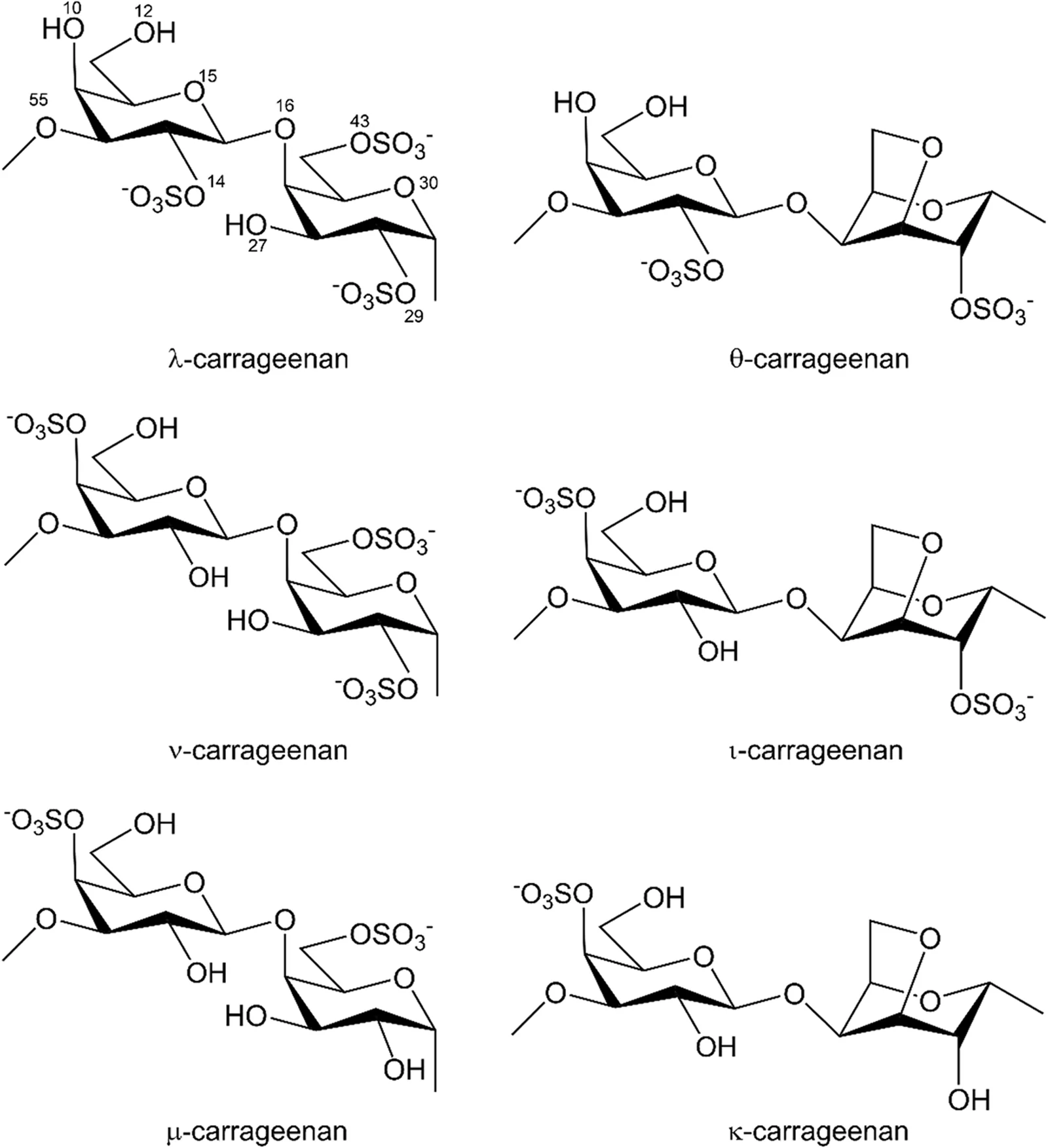
Molecular structures of the six principal types of carrageenans: iota (ι), kappa (κ), lambda (λ), mu (μ), nu (ν), and theta (θ).

### 5.2 Weight Loss Measurements

Carbon steel specimens (API P110) were used in the corrosion tests. The experimental procedure is described in a prior publication^35^ and follows the ASTM G31 standard.^63^ Briefly, the samples were subjected to appropriate surface preparation before immersion and to standardized cleaning procedures after the tests. Immersion experiments were carried out in 1 M HCl over the temperature range 303–333 K and for exposure times of 6, 15, and 24 h. These immersion times have been previously reported in literature.^64–67^ The corrosion behavior was evaluated by comparing the weight loss between the inhibited and blank solutions. The corrosion rate and inhibition efficiency were then determined for each investigated condition.

### 5.3 Biopolymer Characterization—FTIR, TGA, and Kinematic Viscosity

Infrared spectra were obtained by Fourier Transform Infrared Spectroscopy using a PerkinElmer Frontier instrument equipped with an attenuated total reflectance (ATR) module, covering the spectral range from 4000 to 600 cm^–1^. The thermal stability and decomposition profiles of the samples were assessed by thermogravimetric analysis on a TA Instruments Q500 analyzer. Measurements were conducted by heating the samples from 303 to 1123 K at a constant rate of 10 K/min under an inert nitrogen atmosphere flowing at 60 mL/min, using an initial sample mass of approximately 10 mg. In addition, the kinematic viscosities of 1 M HCl and of the acidic solution containing 1500 ppm of the biopolymer were measured with a Stabinger-type automatic viscometer (Anton Paar, SVM3000), following the ASTM D7042–16E1 procedure.^68^

### 5.4 Surface Analysis–Scanning Electron Microscopy (SEM-EDX)

The surface morphology and elemental composition of the API P110 carbon steel samples were characterized using scanning electron microscopy (SEM) with a TESCAN Vega microscope equipped with an energy-dispersive X-ray (EDX) detector. Prior to SEM analysis, gravimetric corrosion experiments were carried out on steel coupons, both without inhibitor and with 1500 ppm inhibitor, at 333 K for an exposure period of 6 h.

### 5.5 Electrochemical Measurements

#### 5.5.1 Static Conditions

Electrochemical tests were performed in triplicate on a PalmSense4 Potentiostat Impedance Analyzer. The system consisted of a conventional three-electrode 400 mL glass cell at 303 ± 1 K, featuring a silver/silver chloride (Ag/AgCl) reference electrode, a platinum counter electrode, and a carbon steel (API P110) working electrode (WE) with an exposed surface area of 1 cm^2^. The working electrode was abraded with 220, 400, and 600 mesh emery paper. The open-circuit potential (OCP) for API P110 carbon steel was monitored for 2 h prior to the measurements during which the electrode potential stabilized to a quasi-stationary value, which was considered the corrosion potential (*E*_corr_). Linear polarization resistance (LPR) tests were then performed and repeated at 4 h intervals, followed by potentiodynamic polarization (PDP). LPR was performed by polarizing the working electrode from −15 to +15 mV in relation to *E*_corr_, at a scan rate of 0.333 mV/s. PDP measurements were performed by scanning the potential from −250 to +250 mV relative to *E*_corr_ at the same scan rate (0.333 mV/s). The anodic (β_a_) and cathodic (β_c_) Tafel slopes were determined by Tafel extrapolation. The polarization resistance (R_p_) was determined from the slope of the potential versus current density curve within the ±15 mV polarization range. No IR compensation was applied during the electrochemical measurements. The electrolyte was 1 M HCl, which has high ionic conductivity. Inhibition efficiencies derived from LPR and PDP data were calculated according to ref 35 by comparing *R*_p_ and corrosion current density (*i*_corr_) values obtained in inhibited and uninhibited (blank) solutions, respectively. For comparison, the corrosion current density was also estimated from the polarization resistance (*R*_p_) using the Stern–Geary eq 12

12 $$i_{\mathrm{corr}}=\frac{|\beta_{a}|\times \left|\beta_{c}\right|}{2.303\left(\left|\beta_{a}\right|+\left|\beta_{c}\right|\right)\times R_{p}}$$

#### 5.5.2 Hydrodynamic Conditions

Electrochemical tests under hydrodynamic conditions (500 and 1500 rpm) were performed using a rotating disk configuration, as an extension of the previously described static measurements. A cylindrical API P110 carbon steel rotating disk electrode with an exposed area of approximately 0.77 cm^2^ was employed, with rotation controlled by a motorized system. After OCP stabilization for up to 2 h, LPR and PDP measurements were carried out, in triplicate.

### 5.6 Molecular Modeling

#### 5.6.1 DFT Calculations

The λ-carrageenan molecules were modeled in their ionic form (3 × SO_3_^–^), as well as in their neutral (3 × SO_3_H) and protonated states, employing Density Functional Theory (DFT) within the gas phase. Calculations were carried out using the B3LYP exchange–correlation functional combined with the 6–31G­(d,p) basis set, in Gaussian 09 software package.^69^ From these computations, quantum-chemical descriptors were determined, including the energies of the highest occupied (*E*_HOMO_) and lowest unoccupied (*E*_LUMO_) molecular orbitals, the energy gap (Δ*E*), chemical hardness (η), and softness (σ).

#### 5.6.2 Molecular Modeling

The interaction of the neutral λ-carrageenan molecule (1500 ppm, 1 M HCl) with the metal was investigated using a molecular model for 303, 318, and 333 K. The simulated system, shown in Figure 13, consisted of one λ-carrageenan molecule, 396 hydronium ions (H_3_O^+^), 396 chloride ions (Cl^–^), 22,073 water molecules, and four layers of iron, each containing 1024 atoms. The iron layers were set to a rigid body during the simulation.

All molecular dynamics simulations were performed using the 2024.3 software^70^ employing periodic boundary conditions in the three spatial directions and an integration time step of 2 fs. All calculations were conducted using all-atom force fields. The all-atom OPLS-AA force field^71^ is widely employed in molecular dynamics studies of potential corrosion inhibitors interacting with metallic surfaces.^72–74^ For water, the TIP4P model was used.^75^ The nonbonded potential parameters for iron atoms were taken from previously validated literature data.^76^ Nonbonded parameters for iron atoms were taken from validated literature sources. Short-range van der Waals interactions were treated using a Lennard-Jones potential with a switching function between 1.1 and 1.3 nm. In comparison, long-range electrostatic interactions were computed using the Particle-Mesh Ewald (PME) method with a real-space cutoff of 1.3 nm.^77^ All nonbonded interactions were truncated at 1.5 nm.

Bond lengths involving hydrogen atoms were constrained via the LINCS algorithm.^78^ The system was first equilibrated in the NVT ensemble for 20 ns at 303 K and 1 bar. Subsequently, production simulations were carried out for a total of 150 ns, divided into three stages of 50 ns each at 303, 318, and 333 K, respectively. Temperature control was maintained using the V-rescale^79^ thermostat with a coupling constant of 1 ps^–1^ (Figure 14).

**Figure 14:**
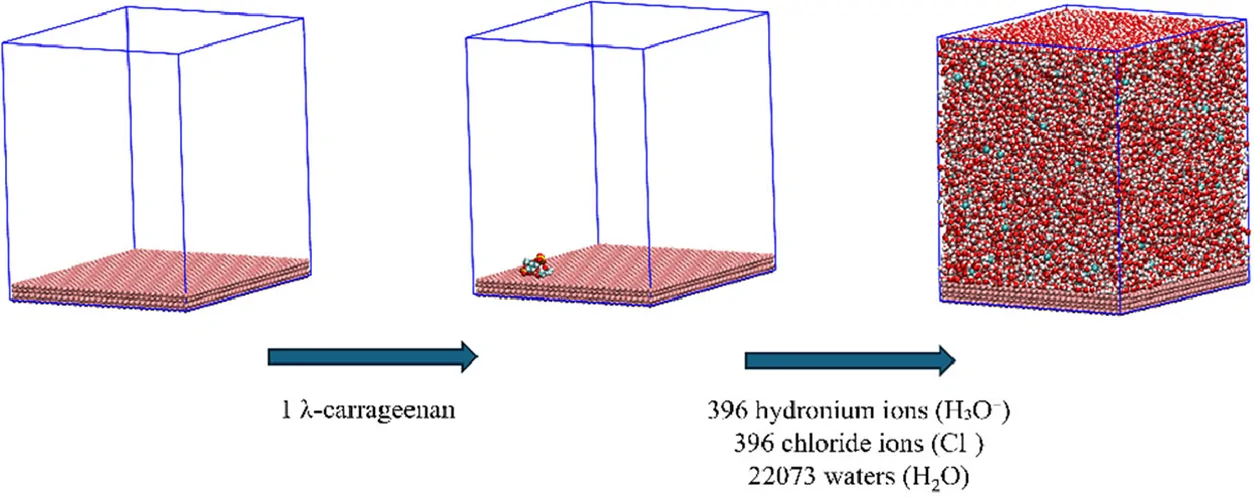
All-atom molecular model of λ-carrageenan at a concentration of 1500 ppm in 1 M HCl solution interacting with an iron surface. The figure shows the complete simulation box containing λ-carrageenan, hydronium ions (H_3_O^+^), chloride ions (Cl^–^), water molecules, and the metallic Fe(110) surface. The inhibitor and solvent molecules are homogeneously distributed within the simulation box. Atom color scheme: cyan = carbon, red = oxygen, yellow = sulfur, white = hydrogen, and pink = iron.
